# Solid dispersion systems for enhanced dissolution of poorly water-soluble candesartan cilexetil: In vitro evaluation and simulated pharmacokinetics studies

**DOI:** 10.1371/journal.pone.0303900

**Published:** 2024-06-06

**Authors:** Israa Saad Mustafa Ali, Uday Aziz Sajad, Bazigha K. Abdul Rasool

**Affiliations:** 1 Pharmaceutics Department, College of Pharmacy, University of Basrah, Basrah, Iraq; 2 Pharmaceutics Department, College of Pharmacy, Almaaqal University, Basrah, Iraq; 3 Department of Pharmaceutical Sciences, Dubai Pharmacy College for Girls, Dubai, United Arab Emirates; Egaz Moniz School of Health and Science, PORTUGAL

## Abstract

**Background:**

Candesartan cilexetil (CC) is a selective angiotensin II receptor antagonist widely used to treat hypertension. CC is a substrate of P-glycoprotein (P-gp), causing its efflux to the intestinal lumen. It is also practically insoluble in water and has low oral bioavailability (14%). Thus, the current study aims to improve the *in vitro* dissolution of CC by developing solid dispersion systems (SDSs) and corroborating the *in vitro* results using a simulated pharmacokinetics study.

**Methods:**

The SDSs were prepared using polyvinyl pyrrolidone (PVP) as a water-soluble polymer, Eudragit E100 (EE100) as a pH-dependent soluble carrier, and a combination of these two polymers. The saturation solubility and the dissolution rate studies of the prepared systems in three dissolution media were performed. The optimized system SE-EE_5_ was selected for further investigations, including DSC, XRD, FTIR, FESEM, DLS, TSEM, IVIVC convolution study, and stability studies.

**Results:**

The solubility of CC significantly increased by a factor of 27,037.344 when formulated as a solid dispersion matrix using EE100 at a ratio of 1:5 (w/w) drug to polymer (SE-EE5 SD), compared to the solubility of the pure drug. The mechanism of solubility and dissolution rate enhancement of CC by the optimized SDS was found to be via the conversion of the crystalline CC into the amorphous form as well as nanoparticles formation upon dissolution at a pH below 5. The instrumental analysis tests showed good compatibility between CC and EE100 and there was no chemical interaction between the drug and the polymer. Moreover, the stability tests confirmed that the optimized system was stable after three months of storage at 25°C.

**Conclusion:**

The utilization of the solid dispersion technique employing EE 100 polymer as a matrix demonstrates significant success in enhancing the solubility, dissolution, and subsequently, the bioavailability of water-insoluble drugs like CC.

## 1. Introduction

Oral administration is the preferred and most frequently used route of drug delivery, it is non-invasive, self-administered, does not require special management skills or frequent clinic visits, and is more acceptable to patients. In addition, oral dosage forms are easier to handle and store than the parenteral route and relatively safer because the drug does not pass directly into the blood [1]. The main issue with oral administration is the active pharmaceutical ingredient’s (API) bioavailability which depends on many physiological, pathophysiological, and pharmaceutical factors. For an orally administered drug to be absorbed, it should present in its soluble form at the absorption site [2]. Poorly soluble drugs such as APIs listed in classes II and IV according to the Biopharmaceutics Classification System (BCS) may pass their absorption site before complete dissolution, and will typically exhibit a dissolution rate-limited absorption causing low bioavailability [3].

About 70% of current candidate new drugs have poor water solubility; therefore, several solubility improvement strategies have been investigated over the past few decades to enhance drug solubility and bioavailability. Some of these techniques are size reduction by microparticulate system [4], dendrimers [5], chemical modification, pro-drug, and salt formation, co-crystals, co-solvency, complexation with cyclodextrin, pH modification, self-emulsifying drug delivery system, and solid dispersion systems (SDSs) [6]. Each of these methods has its advantages and limitations. However, solid dispersion technique is one of the most interesting approaches among all these methods. [7] defined the SDSs as "a dispersion of one or more active ingredients in an inert carrier or matrix (hydrophilic) at solid-state prepared by fusion, solvent or melting solvent method". It converts the particles to an amorphous state and reduces particle size, resulting in improved drug particles’ wettability, porosity, and solubility [8]. The solid dispersion techniques also improve drug stability by reducing the aggregation of particles [9] and can be employed to control and prolong drug release [10]. In addition, the method applied demonstrated the ease of preparation and optimization, as well as the reproducibility of the fabrication method [11].

Various types of natural and synthetic polymers were used to fabricate the SDSs such as PVP, chitosan, sodium alginate, cellulose derivatives, and Eudragits. In our study, PVP K30 and Eudragit® E 100 were selected to prepare the CC-SDSs at different weight ratios. PVP K30 is a nonionic amorphous polymer with a molecular weight of 66,800 g/Mol. It is soluble in water, ethanol, and isopropyl alcohol. Also, it is an ideal candidate for spray-dried solid dispersions through the spray drying method, which is an easily scaled-up technique for commercial production [12]. While Eudragit^®^ E 100 is a cationic copolymer that has an average molar mass of approximately 47,000 g/Mol. It is colorless to yellow-tinged granules with a characteristic amine-like odor. EE100 has been widely used in SDSs as a carrier polymer to enhance the solubility and/or bioavailability of drugs, especially in gastric media owing to its good solubility at pH ≤ 5.0 [13].

**Fig 1:**
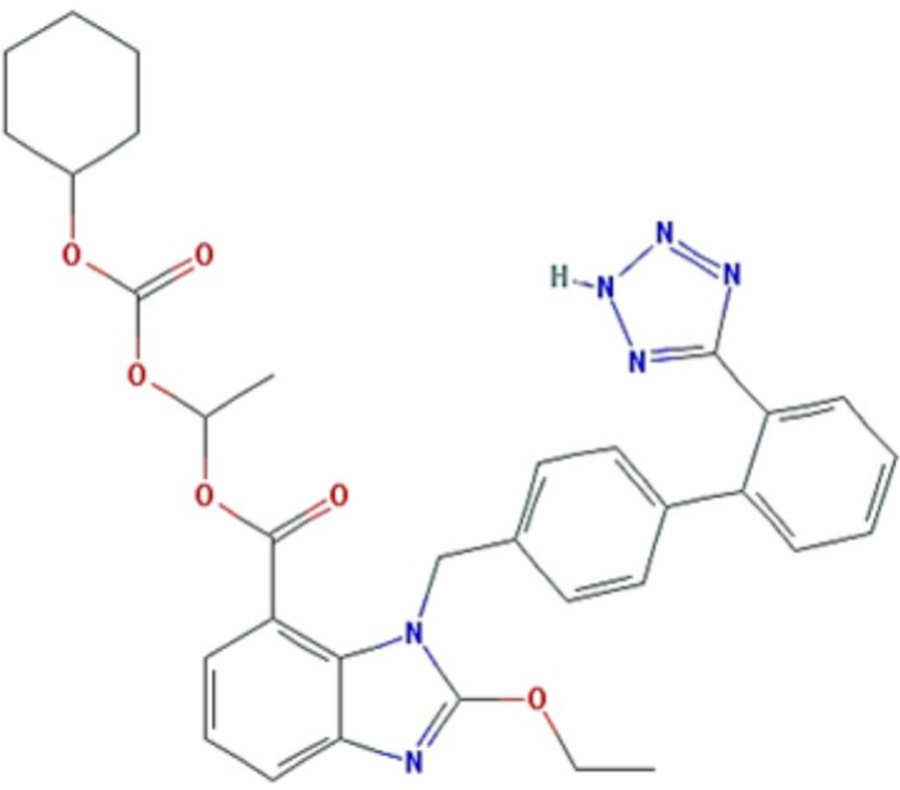
Chemical structure of CC (Cyclohexyl carbonate ester of (±)-1-hydroxyethyl 2-ethoxy-1-[p-(o-1H-tetrazol-5-ylphenyl)benzyl]-7-benzimidazolecarboxylate).

On the other hand, CC is practically insoluble in water (less than 5 × 10^− 5^ mg/mL) and sparingly soluble in methanol [14]. Fig 1 displays the chemical structure of CC. In fact, CC is a prodrug; it is rapidly bioactivated to the active candesartan by ester hydrolysis during absorption from the gastrointestinal tract. It is indicated for the treatment of hypertension and heart failure by blocking the vasoconstrictor-secreting effects of angiotensin II in many tissues such as vascular smooth muscle and the adrenal gland. Following oral administration of CC tablets, the absolute bioavailability of CC is estimated to be approximately 15%, the peak serum concentration (C_max_) is reached after 3–4 hours, and the plasma half-life of candesartan is 9 hrs. The bioavailability of CC isnot affected by food [14]. This study aims to improve the solubility of CC by employing the solid dispersion technique and to investigate the underlying mechanisms responsible for the enhancement of dissolution.

## 2. Materials and methods

### 2.1. Materials

CC powder was purchased from Zhejiang Kinglyuan Pharmaceuticals (China). EE100 polymer from Shanghai Reuzheng Chemical Tech Co., Ltd (China). PVP polymer was from Baoji Guokang Bio-Te1chnology Co., Ltd (China). All the used solvents were of analytical grade.

### 2.2. HPLC quantification of CC

CC was quantified in the prepared SDSs using a reverse-phase autosampler Shimadzu HPLC system (Japan) equipped with a UV detector and LabSolutions software. The separation was achieved on a C8 column, with a diameter of 150 mm × 4.6 mm. The column temperature was 30°C. The elution was under isocratic pressure, with a mobile phase of acetonitrile and water (55: 45 v/v) and 0.1% trifluoroacetic acid (TFA). The running time was 1.8 times the retention time of CC with a flow rate of 1.5 mL/min. The UV detection was at 254 nm and the injection volume was 50 μL [15]. The concentration of CC in the samples was determined using a calibration curve constructed earlier in acetonitrile. Microsoft® Excel sheet 2019 was used to perform a linear regression analysis to determine the linear equation and the correlation coefficient, R^2^ (y = 23.602 x-1.747; R^2^ = 0.9999). A high R^2^ value indicates that the calibration curve was constructed following Beer’s law and the concentrations used were convenient. The procedure of the CC calibration curve’s preparation along with the plotted curve is given in S1 File.

### 2.3. Preparation of CC-SDs and physical mixtures

#### 2.3.1. Kneading method

The kneading method was used in the preparation of PVP-based SDSs. The polymer and the drug were mixed for 10 minutes in a mortar at a specified weight ratio. After kneading with a few drops of ethanol for one hour, the resulting slurry was placed in the hot air oven for 24 hours at 40°C to ensure complete drying. After drying, the samples were ground, sieved in a NO# 70 mesh sieve, placed in screw-cup containers, and stored in a desiccator at 25°C for further studies [16]. The codes of prepared SDSs were symbolized as (KN_1_, KN_3_, and KN_5_), as shown in Table 1.

**Table 1:** Formulation compositions of CC-SDSs.

SDSs codes	PVP (g)	EE100 (g)	Drug: Polymer ratio (*w/w*)	Preparation Technique
KN_1_	2	…	1:1	KN
KN_3_	6	…	1:3	KN
KN_5_	10	…	1:5	KN
SE_1_	2	…	1:1	SE
SE_3_	6	…	1:3	SE
SE_5_	10	…	1:5	SE
SE-EE_1_	…	2	1:1	SE
SE-EE_3_	…	6	1:3	SE
SE-EE_5_	…	10	1:5	SE
SE-CO_1_	2	4	1:1:2	SE
SE-CO_2_	3	3	1:1.5:1.5	SE
SE-CO_3_	4	3	1:2:1.5	SE
SE-CO_4_	4	2	1:2:1	SE

KN: Kneading technique; SE: Solvent evaporation technique; PVP: Polyvinylpyrrolidone; EE 100: Eudragit E100; *w/w*: percentage weigh-in-weight. Each system contains 2 g of CC.

#### 2.3.2. Solvent evaporation method

In the solvent evaporation method, the polymers were dissolved in 100 mL ethanol and sonicated for 15 min. Then CC was added to the polymer solutions and sonicated for 5 minutes, the resulting solutions were poured into a mortar and kept in a hot air oven at 40°C. After 24 h., the resulting mass was ground and sieved in NO# 70 sieve and kept in a screw cap container in a desiccator at 25°C for further studies (Table 1) [17].

#### 2.3.3. Preparation of CC-polymers physical mixtures

An accurately weighed amount of CC and polymers in various drug to carriers’ weight ratios, as specified in Table 1, were thoroughly blended in glass mortar for 15 min. The products were placed in screw-cap containers and stored in a desiccator at 25°C to be used for further investigations [10].

### 2.4. Characterizations of CC-SDSs

#### 2.4.1. Drug content

The accurate weight of the SD powder equivalent to 16 mg of CC was weighed, mixed with a small volume of HPLC grade methanol, and transferred to a 10 mL volumetric flask. Methanol was added up to 10 mL and the resulting solution was sonicated, left to rest, and filtered. Then 0.1 mL was withdrawn and diluted with methanol up to 10 mL, then the concentrations were measured by an autosampler HPLC system (Shimadzu, Japan). The drug content was measured with Eq 1 [18]. The results were given as a mean ± SD (n = 3).


Drugcontent(%)=(Drugweight)/(WeightofpreparedSD)×100 Eq 1

#### 2.4.2. Characterization of CC-SDSs powder flowability

The flow properties of the powder samples were assessed based on determining the angle of repose, bulk density, tapped density, Carr’s compressibility index, and Hausner ratio (HR). The results were given as a mean ± SD (n = 3).

*Angle of repose*. The angle of repose of the powder blend was determined by the fixed funnel method using the Copley Scientific funnel. A specified weight (2 g) of each powder sample was poured into the funnel. Then the powder was allowed to flow freely on the plain surface. The height and the diameter of the formed cone were measured after all of the powder in the funnel was evacuated. The angle of repose was calculated using Eq 2 [19]:

Tanθ=h/r Eq 2

Where; h and r are the height and radius of the powder’s cone, respectively. The results were given as a mean ± SD (n = 3).

*Bulk density and tapped density*. Both bulk density (BD) and tapped density (TD) were determined. A quantity of 2 g of powder blend from each SDS was introduced into a 10 mL measuring cylinder attached to the Copley^®^ apparatus. After that, the initial volume was recorded and the bulk density was measured by Eq 3. The apparatus was operated at 100 taps per min. for 5 min. and the tapped density was calculated using Eq 4 [19]:

BD=(Weightofthepowder)/(Untappedvolumeofthepowder) Eq 3

TD=(Weightofthepowder)/(Tappedvolumeofthepowder) Eq 4

*Determination of Carr’s compressibility index and Hauser ratio*. The bulk and tapped densities were used to calculate Carr’s compressibility index and Hausner’s ratio to assess the flow properties and compressibility of the prepared CC-SDSs. The obtained measurements were compared to a standard reference to judge the flow properties of powder. As shown in Eqs 5 and 6 [19]:

$$Carr^{\prime }s\;Index=((TD-BD)/TD\times 100$$ Eq 5

Hausnerratio=(TD)/(BD) Eq 6

Where; TD and BD are the tapped and bulk density, respectively.

### 2.5. Saturation solubility

The saturation solubility was measured in 0.1 N HCl pH 1.2, acetate buffer pH 4.5, and phosphate buffer pH 6.5, separately. In 100 mL volumetric flasks, an excess amount of CC powder, CC-polymers physical mixture, and CC-SDSs were added to 10 mL of each dissolution media. All the samples were shaken at 100 rpm in a shaking water bath (Nuve ST 402, UK) at 25°C. After 72 h., the samples were centrifuged for 30 minutes in a cooling centrifuge at 25°C and 6000 rpm. From the filtrate, 0.1 mL was withdrawn by syringes equipped with a 0.45 μm pore size filter syringe and diluted to 10 ml by the dissolving media. The drug concentration was measured by the HPLC system (Shimadzu, Japan). The saturated solubility study was performed in triplicate and calculated as a mean± SD. Eq 7 was used to compute the solubility enhancement ratio of CC in the form of SDS compared to the saturated solubility of CC in the acidic and alkaline media [20]:

$$Solubility\;Enhancement\;Ratio\;(ER)=\frac{CC-Solubility\;(SDS)}{CC-Solubility\;(pure\;drug)\;}$$ Eq 7

### 2.6. *In vitro* dissolution studies

The *in vitro* dissolution studies of the prepared SDSs and the pure CC were carried out at 37± 0.5°C and 50 rpm using 900 mL of: 0.1 N HCl pH 1.2, phosphate buffer pH 6.5, and acetate buffer pH 4.5 in the paddle type II USP dissolution apparatus under sink condition. Accurately weighed samples equivalent to 16 mg of CC were added to each dissolution media. Samples (5 ml) were withdrawn by a syringe equipped with a filter syringe of 0.45 μm pore size and replaced with an equal volume of fresh dissolution medium at time intervals of 15, 30, 45, 60, 90, 120, and 180 minutes. The withdrawn samples were analyzed using an autosampler HPLC system (Shimadzu, Japan), and the injection volume was 50 μL [21]. The tests were performed in triplicate (n = 3) and the results were given as a mean ± SD.

### 2.7. Simulated pharmacokinetics study

#### Prediction of *in vivo* data

The simulated drug plasma concentration vs. time profile of CC was predicted from the *in vitro* release data of the optimized formula (SE-EE_5_) by utilizing the convolution method [22]. The cumulative drug release (%) values obtained from the drug *in vitro* dissolution data were converted into distinct amounts (mg) at each sampling time point. For each segment of drug amount, the quantity of drug eliminated was calculated using the first-order elimination rate constant which was obtained from Eq 8:

Ke=0.693/t1/2 Eq 8

Where Ke: is the first-order elimination rate and t_1/2_ is the half-life of the drug.

In the following step, drug amounts at different time intervals were added to determine the total amount present in the blood. Finally, the plasma concentration of CC (μg/mL) was computed by multiplying the blood amount of each time interval by the drug bioavailability and then dividing it by the drug volume of distribution (Eq 9):

Conc.=Amt*F*1000/Vd Eq 9

Where; Conc. is the drug concentration in the blood (μg/mL) at t time, F: is the bioavailability factor, Vd: is the volume of distribution, and 1000: is the conversion factor to report concentration in ng/mL.

#### Pharmacokinetics parameters of CC

The pharmacokinetic parameters of CC were obtained from published papers with a high level of authenticity [23] where the bioavailability of CC is 15%, the half-life is 9 hrs., and the Vd is 0.13 L/kg. However, other parameters, *C*_max_, *T*_max_, and *AUC* were calculated depending on the predicted *in vivo* data.

#### *In vitro*–*in vivo* correlation (IVIVC) study

The level-A *in vitro*-*in vivo* correction was constructed by plotting *in vitro* drug release data (%) *versus* the simulated plasma drug concentration (ng/mL). The plasma concentration-time intervals were started at 0 h and ended at 24 h. The linear regression coefficient (*R*^2^) was obtained and compared between the optimized formula SE-EE_5_ and the commercial CC tablet for efficient *in vivo* performance.

### 2.8. Differential scanning calorimetric (DSC)

The DSC studies were performed to evaluate the thermal profile for the pure drug, polymer (EE100), CC-polymer physical mixture, and selected CC-SD system. The powders were transferred into an aluminum pan and tested. In this work, Shimadzu DSC-60A Plus (Japan) with LabSolution software was used. The heating range was from 30 to 300°C at an increasing heating rate of 10°C/min.

### 2.9. X-ray diffraction measurements (XRD)

The powder X-ray diffractograms of pure drug, polymer (EE100), CC-polymer physical mixture, and the selected CC-SD system were characterized by Xpert-pro PANanalytical (Holand). The tested powders were pressed into disks. The instrument was operated at 20 mA, 40 kV using a Copper anode as an X-ray source. Diffraction patterns were recorded over the 2-theta range of 8–80°C [24].

### 2.10. Fourier Transform Infrared (FT-IR) spectroscopy

KBr disks of pure drug, polymer (EE100), CC-polymer physical, and the selected CC-SD system were prepared for FTIR analysis to study the interactions between the API and the excipients. The KBr powder was mixed with the powders to be analyzed in a mortar and compressed into 1–2 mm thickness discs. Shimadzu FTIR 4200 (Japan) was used. The scanning wavelength ranged from 400 cm^-1^ to 4000 cm^-1^.

### 2.11. Field emission scanning electron microscopic (FE-SEM) study

To study the surface and morphology of the pure drug, polymer (EE100), CC-polymer physical mixture, and the selected CC-SD system, samples were loaded on a specimen stub using double-side carbon tape. They stuck down the mask layer on it, then tight all stubs on the specimen’s holder after blowing to remove any non-adherent particles. The prepared samples were loaded on FE-SEM from Zeiss/Supra 55 VP (Germany) [25].

### 2.12. Dynamic light scattering (DLS) analysis

To detect the presence of nanoparticles, two milliliters of 2 mg/mL SE-EE_5_ solution in 0.1 HCl were poured into a disposable cuvette and tested by a zeta sizer (NanoZS, Malvern, UK). The average size, size distribution, and polydispersibility index (PDI) were measured.

### 2.13. Transition scanning electron microscopy (TSEM) analysis

A TSEM test was done to confirm the nanoparticles’ spherical shape. The solution of 2 mg/mL of SE-EE_5_ was spread on a copper grid previously covered with a carbon layer then the grid was transferred to the STEM (Nova NanoSEM, Holand) and tested.

### 2.14. Stability study

The optimized system SE-EE_5_ was stored at an ambient temperature in a desiccator for 12 months. At the end of the storage duration, the powder was examined for organoleptic properties, FTIR spectroscopy analysis, XRD analysis, drug content, and *in vitro* drug dissolution study to evaluate the stability of the drug upon storage and aging.

### 2.15. Statistical analysis

The statistical analysis was conducted using IBM SPSS^®^ Statistics software, version 28.0.1.0, employing One-way ANOVA to ascertain statistically significant differences at a *p*-level of 0.05. Results were presented as mean values ± SD (n = 3).

## 3. Results and discussion

### 3.1. HPLC quantification of CC

**Fig 2:**
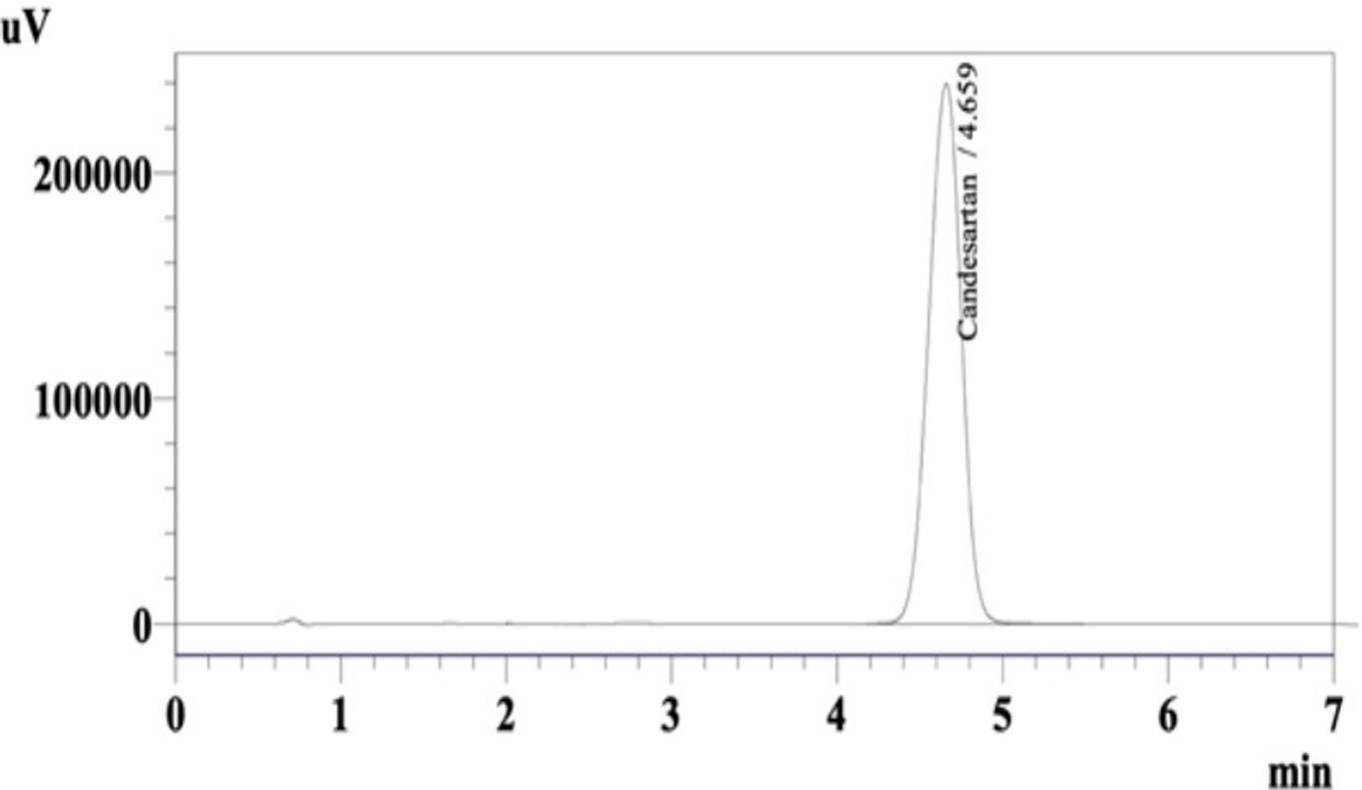
HPLC chromatogram of CC in acetonitrile.

After setting the autosampler HPLC system to inject 50 μL of 0.35 mg/mL of CC solution in acetonitrile, a sharp peak was obtained for CC with a retention time of 4.659 minutes (Fig 2). The analysis was repeated six times (n = 6).

### 3.2. Preparation of CC-SDSs

CC-SDSs were prepared using solvent evaporation and kneading techniques. Although both methods are well-established and widely documented in prior literature, our findings however showed the greater reliability and reproducibility of the solvent evaporation compared to kneading. In addition, the SDSs were formulated by incorporating the drug into the polymer matrix in three drug-to-polymer weight ratios including 1:1, 1:3, and 1:5. The ratios’ selection reflected a deliberate approach aimed at optimizing the formulation’s performance by encompassing a diverse range of ratios to explore their influence on the properties of the systems. Our prior experience with similar formulations indeed provided valuable insights into the ratios’ selection as well as their potential impact on the drug solubility and the system’s characteristics [10].

### 3.3. Characterization of CC-SDSs

#### 3.3.1. Drug content

The systems prepared via the kneading method exhibited the lowest drug content, attributed to the method’s inability to consistently disperse the drug within the polymer matrix (Table 2). Notably, systems prepared using EE100 polymer alone demonstrated higher drug content than other systems.

**Table 2:** Characteristics of the prepared SDSs.

CC-SDSs	Drug content (%)	Hausner’s ratio	Carr’s index (%)	Angle of repose (°)	Flowability
KN_1_	95.09 ± 0.36	1.02 ± 0.2	8.85 ± 0.09	26.03 ± 0.12	Excellent
KN_3_	95.93 ± 0.13	1.10 ± 0.01	5.95 ± 0.44	27.98 ± 0.03	Excellent
KN_5_	96.21 ± 1.54	1.07 ± 0.02	7.96 ± 0.43	27.39 ± 0.23	Excellent
SE_1_	98.49 ± 0.62	1.04 ± 0.02	7.96 ± 0.33	28.66 ± 0.34	Excellent
SE_3_	99.01 ± 0.25	1.06 ± 0.03	7.94 ± 1.03	26.04 ± 1.02	Excellent
SE_5_	98.30 ± 1.05	1.07 ± 0.03	5.54 ± 0.03	27.63 ± 0.46	Excellent
SE-EE_1_	99.45 ± 0.93	1.03 ± 0.01	7.5 ± 0.04	29.00 ± 0.54	Excellent
SE-EE_3_	99.12 ± 0.08	1.05 ± 0.04	8.22 ± 0.77	28.32 ± 0.39	Excellent
SE-EE_5_	99.09 ± 0.90	1.05 ± 0.03	6.04 ± 0.23	27.65 ± 0.45	Excellent
SE-CO_1_	98.20 ± 1.23	1.08 ± 0.01	6.65 ± 0.26	25.89 ± 0.35	Excellent
SE-CO_2_	97.99 ± 0.33	1.07 ± 0.02	7.12 ± 0.19	26.77 ± 0.52	Excellent
SE-CO_3_	98.20 ± 0.24	1.04 ± 0.03	7.91 ± 0.26	28.29 ± 0.62	Excellent
SE-CO_4_	96.04 ± 1.90	1.04 ± 0.02	6.66 ± 0.55	27.61 ± 0.44	Excellent

#### 3.3.2. Characterization of CC-SDSs powder flowability

The flowability study results are depicted in Table 2. The solid dispersion formulations demonstrated excellent flowability, indicating their suitability for incorporation into various dosage forms.

### 3.4. Saturation solubility studies

The solubility of pure CC, the physical mixture of polymers and CC, and CC-SDSs in the simulated gastric (pH 1.2), small intestine (pH 4.5), and large intestine (pH 6.8) media are presented in Tables 3–5, respectively. These results indicated an increase in the solubility of CC when prepared as solid dispersion compared with the pure drug and the physical mixture of the drug and the polymers. While CC is practically insoluble in aqueous media, all the prepared systems showed an improvement in solubility to a specific extent.

**Table 3:** Saturation solubility and solubility enhancement ratio of CC, CC-polymers physical mixtures, and CC-SDSs in HCl (pH 1.2).

Formulae codes	CC-SDSs (mg/mL)	CC-polymers physical mixture (mg/mL)	Enhancement ratio	
			CC-SDSs	Physical mixtures
KN_1_	0.010 ± 0.005	0.003±0.003	41.493	12.448
KN_3_	0.014 ± 0.009	0.004±0.001	58.091	16.597
KN_5_	0.017 ± 0.003	0.005±0.002	70.539	20.746
SE_1_	0.050 ± 0.008	-	207.468	-
SE_3_	0.140 ± 0.102	-	580.912	-
SE_5_	0.152 ± 0.042	-	630.705	-
SE-EE_1_	1.277 ± 0.577	0.007 ± 0.010	5298.755	29.045
SE-EE_3_	5.311 ± 0.345	0.028 ± 0.033	22037.344	116.182
SE-EE_5_	6.516 ± 0.508	0.033 ± 0.052	27037.344	136.929
SE-CO_1_	1.813 ± 1	0.005 ± 0.002	7522.821	20.746
SE-CO_2_	5.264 ± 1.523	0.014 ± 0.020	21842.323	58.091
SE-CO_3_	5.564 ± 1.619	0.007 ± 0.001	23087.136	29.045
SE-CO_4_	4.009 ± 1.527	0.004 ± 0.009	16634.854	16.597
CC	0.00024 ± 0.0002			

**Table 4:** Saturation solubility and solubility enhancement ratio of CC, CC-polymers physical mixtures, and CC-SDSs in acetate buffer (pH 4.5).

Formulae codes	CC-SDSs (mg/mL)	CC-polymers physical mixture (mg/mL)	Enhancement ratio	
			CC-SDSs	Physical mixtures
KN_1_	0.001 ± 0.003	0.003 ± 0.004	4.149	12.448
KN_3_	0.003 ± 0.051	0.003 ± 0.001	12.448	12.448
KN_5_	0.007 ± 0.055	0.006 ± 0.009	29.045	24.896
SE_1_	0.012 ± 0.011	-	49.792	-
SE_3_	0.013 ± 0.031	-	53.941	-
SE_5_	0.015 ± 0.083	-	62.240	-
SE-EE_1_	0.903 ± 0.700	0.008 ± 0.002	3746.887	33.195
SE-EE_3_	5.201 ± 1.050	0.035 ± 0.003	22037.344	116.182
SE-EE_5_	6.550 ± 1.639	0.062 ± 0.050	27037.344	136.929
SE-CO_1_	1.943 ± 0.299	0.003 ± 0.002	8062.240	12.448
SE-CO_2_	2.339 ± 1.009	0.016 ± 0.020	9705.323	66.390
SE-CO_3_	3.954 ± 1.001	0.007 ± 0.003	16406.639	29.045
SE-CO_4_	0.010 ± 0.002	0.003 ± 0.007	41.493	12.448
CC	0.00024 ± 0.0002			

**Table 5:** Saturation solubility and solubility enhancement ratio of CC, CC-polymers physical mixtures, and CC-SDSs in phosphate buffer (pH 6.8).

Formulae codes	CC-SDSs (mg/mL)	CC-polymers physical mixture (mg/mL)	Enhancement ratio	
			CC-SDSs	Physical mixtures
KN_1_	0.125 ± 0.091	0.005 ± 0.004	471.698	18.867
KN_3_	0.597 ± 0.172	0.006 ± 0.002	2252.830	22.641
KN_5_	0.648 ± 0.213	0.010 ± 0.032	2445.283	40.754
SE_1_	0.552 ± 0.242	-	2083.018	-
SE_3_	2.353 ± 1.009	-	8879.245	-
SE_5_	2.756 ± 0.942	-	10400.210	-
SE-EE_1_	0.004 ± 0.003	0.0002 ± 0.0001	15.094	0.826
SE-EE_3_	0.095 ± 0.004	0.0002 ± 0.003	358.490	0.852
SE-EE_5_	0.097 ± 0.033	0.0004 ± 0.0008	366.037	1.630
SE-CO_1_	0.173 ± 0.009	0.001 ± 0.007	652.830	3.773
SE-CO_2_	0.156 ± 0.123	0.002 ± 0.009	588.679	7.547
SE-CO_3_	0.150 ± 0.005	0.001 ± 0.027	566.037	3.773
SE-CO_4_	0.003 ± 0.019	0.003 ± 0.041	11.320	11.320
CC	0.00026 ± 0.0005	-	-	-

However, in the acidic media (pH = 1.2) (Table 3) and acetate buffer solution (pH = 4.5) (Table 4) the systems that were prepared by kneading methods using PVP showed the least improvement in CC saturation solubility (enhancement ratio is 41.493, 58.091, 70.539 fold in acidic medium and 207.468, 630.705, 580.912 fold in acetate medium for KN_1_, KN_3_, and KN_5_, respectively) compared with other systems indicating the insufficiency of the kneading method to improve CC solubility compared with the solvent evaporation method. This could be attributed to the incomplete dispersion of CC within the polymer matrix in the kneading method as it solely relies on manual pressure at variable rates, proving inefficient for achieving the amorphization of the drug particles [16]. However, the systems that were prepared by the solvent evaporation method using PVP alone as a polymer showed better improvement in saturation solubility than the SDSs that were prepared by the kneading method. This may be attributed to the controlled evaporation of the solvent leading to the formation of uniformly distributed small particles, in which the drug is coated by the polymer resulting in a decrease of crystallinity or an increase in amorphization of the drug [26]. For these systems, the solubility increased as the polymer-to-drug ratio increased, thus SE_5_ exhibited a higher solubility enhancement value than SE_3_, and the least results were seen for SE_1_ (the enhancement ratio is 207.468, 580.912, and 630.705 in the acidic medium and 49.792, 53.941, and 62,240 in the acetate medium for SE_1_, SE_3_, and SE_5_, respectively.

Furthermore, the systems that were prepared by the solvent evaporation method using EE100 polymer only showed the best improvement in the saturation solubility and CC solubility increased with an increase in the polymer-to-drug ratio with higher results obtained from the SE-EE_5_ (the enhancement ratio is 5298.755, 22037.344, and 27037.344 in acidic medium and 3746.887, 21580.912, 27178.423 in acetate medium for SE-EE_1_, SE-EE_3_, SE-EE_5_, respectively) indicating that the amphiphilic polymer EE100 is better than PVP in improving CC solubility at pH = 1.2 and pH = 4.5. The reason for this could be the ionizable nature of the EE100 polymer in the acidic media and this feature could be exploited to enhance the solubility of the weak acidic drug in the upper part of the GIT either to obtain a fast-release system or to prepare gastroretentive drug delivery systems [27].

For the ternary systems prepared by a combination of PVP and EE100, the improvement in CC solubility was lower than that noticed in SE-EE_5_ (the enhancement ratio is 7522.821, 21842.323, 23087.136, and 16634.854 in acidic and 8062.240, 9705.394, 16406.639, and 41.493 in acetate for SE-CO_1_, SE-CO_2_, SE-CO_3_, and SE-CO_4_ respectively). Although PVP acts as a precipitation inhibitor at low polymer concentrations, however at higher concentrations, it may cause drug precipitation due to its influence on drug diffusion from the particle surface to the bulk dissolution medium. This can have a detrimental effect on drug dissolution, as observed in the case of the systems SE-CO_1_, SE-CO_2_, SE-CO_3_, and SE-CO_4_ [28].

Regarding the basic medium (pH = 6.5) (Table 5) the systems prepared by the kneading method with PVP polymer showed an enhancement in solubility of CC (enhancement ratio is 471.698, 2252.830, 2445.283 for KN_1_, KN_3_, KN_5_, respectively) but still less than that of the systems that prepared by the solvent evaporation method due to inadequacy of the kneading method in the preparation of CC SD systems as mentioned by Khan *et al*. [11].

The systems SE_1_, SE_3_, and SE_5_ demonstrated significant (*p*< 0.05) improvement in solubility in the basic medium (pH = 6.5) with enhancement ratios of 2083.018, 8879.245, and 10400, respectively. This enhancement was not observed in acidic or acetate media, possibly due to the ionization of the weak acidic drug following treatment with the appropriate polymer concentration. The increased CC solubility observed in the basic medium suggests that formulating the SDSs for a weakly acidic drug could offer advantages in targeting the distal part of the GIT and facilitating colonic delivery. For the systems SE-EE_1_, SE-EE_3_, and SE-EE_5_ the solubility of CC enhanced by 15.094, 358.490, and 366.037, respectively, but was less than the solubility of the PVP-only based systems because the EE100 polymer is not soluble but still permeable in the basic media [29].

For the systems, SE-CO_1_, SE-CO_2_, SE-CO_3_, and SE-CO_4_ the enhanced solubility (enhancement ratio is 652.830, 588.679, 566.037, and 11.320, respectively) is less than that for the systems prepared by PVP polymer alone. The decrease in solubility became notably more significant (*p*< 0.001) with elevated concentrations of PVP, as evidenced by the SE-CO_4_ formula. This phenomenon could arise from the precipitating effect induced by higher PVP concentrations, as discussed earlier in this section, or it could be attributed to the insolubility of EE100 in a basic medium. Also, the presence of two polymers with CC in the alcoholic medium during preparation may result in a hydrogen bond formation between the two hydrophilic polymers leaving CC unbounded. In the binary systems, the characteristic peaks of CC for both the physical mixture and the system SE-CO_3_ indicated that not all the CC is converted into an amorphous form (Figs 19 and 20).

The solubility enhancement of the physical mixtures for all the systems in the three media was much less than that for the corresponding prepared SD systems which could be explained by the fact that the amorphous state of the drug offers a lower thermodynamic barrier to solubility and the formation of a glassy solution where the drug is molecularly dispersed in the polymer i.e., solubility enhancement is the result of the disordered structure of the amorphous solid. So, because of the short-range intermolecular interactions in an amorphous system, no lattice energy has to be overcome, whereas, in the crystalline material, the lattice has to be disrupted for the material to dissolve [30].

Furthermore, all the prepared systems exhibited a significant enhancement in the saturation solubility in the acidic media (*p*< 0.05) with the SE-EE_5_ SDS exhibiting a highly significant solubility enhancement (*p*< 0.001). Nevertheless, the overall solubility of CC in all the prepared SDSs in phosphate buffer was lower than in the other two media. This could be attributed to the formation of phosphate aggregates in the buffer media as elaborated by K S NS *et al*. who observed the lower solubility of raloxifene HCl in phosphate media and further complexes formation with quercetin which was determined by investigating the residual particles from solubility studies [31].

### 3.5. *In vitro* dissolution studies

The dissolution studies for the plain CC, commercial CC tablets, and the prepared CC-SD systems were performed by using the paddle-type USP dissolution apparatus. The dissolution study was performed to investigate the effect of formulating CC as an SDSs on the dissolution rate of the parent drug. The dissolution studies were done in acidic media (0.1N) HCl (pH = 1.2) to simulate the gastric medium, acetate buffer (pH = 4.5) to simulate the upper part of the small intestine, and phosphate buffer (pH = 6.5) simulating the lower part of the small intestine.

**Fig 3:**
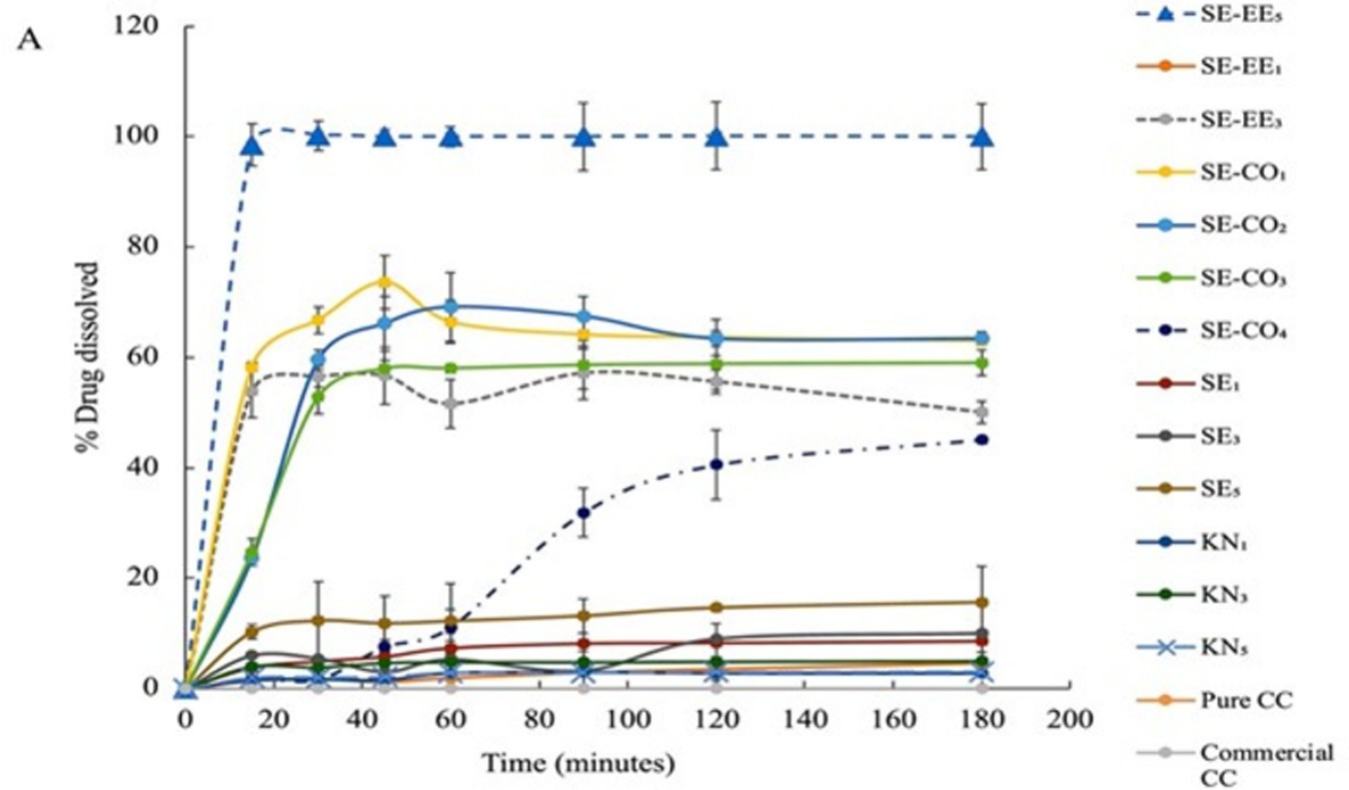
Dissolution profiles of pure CC, commercial CC tablet, and the prepared SDSs in HCl (pH 1.2).

**Fig 4:**
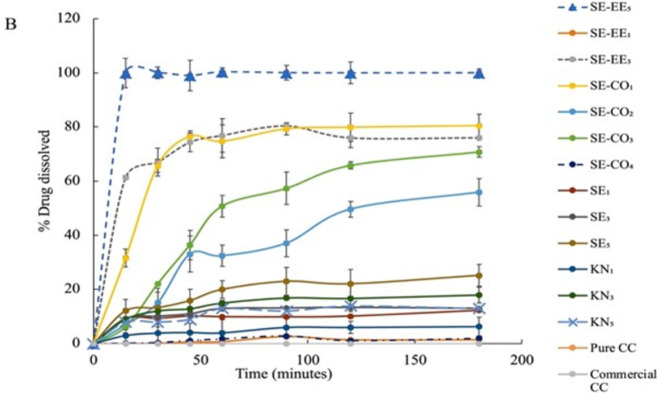
Dissolution profiles of pure CC, commercial CC tablet, and the prepared SDSs in acetate buffer (pH 4.5).

In the acidic medium with 0.1N HCl (pH = 1.2) and the acetate buffer (pH = 4.5) (Figs 3 and 4), the systems prepared by the kneading method with PVP as the carrier polymer (KN_1_, KN_3_, and KN_5_) showed the least improvement in dissolution profile compared with other systems this could be explained by the insufficiency of kneading method to disperse CC evenly within PVP and not all the drug was converted into the amorphous form. Also, the improvement of the dissolution rate was not consistent with the drug: polymer ratio in the kneading method i.e., increasing the polymer-to-drug ratio does not necessarily increase the dissolution rate. The order of improvement in the dissolution profile was KN_1_ < KN_5_ < KN_3_. This could be the result of the insufficient distribution of the CC within the polymer matrix leading to incomplete interaction between the drug and the polymer in the kneading method, so the drug is not dispersed at a molecular and at higher polymer content the PVP acts as a precipitator and can affect the drug diffusion from the particle surface to the bulk medium [28], causes an increase in viscosity of the dissolution medium which obstructs the release of the drug from the powder particles to increase according to the Noyes-Whitney equation (Eq 10) [32]:

dm/dt=DA(Cs-C)/h Eq 10

Where; dm / dt is the rate of dissolution of the drug particles, D: is the diffusion coefficient of the drug in solution in the dissolution medium and is related, in part, to the viscosity of the medium, which will decrease with increasing medium viscosity and decreasing dissolution rate. A: is the effective surface area of the drug particles in contact with the dissolution medium, h: is the thickness of the diffusion layer around each drug particle, Cs: is the saturation solubility of the drug in solution in the diffusion layer, and C: is the concentration of the drug in the dissolution medium.

Consequently, for the systems prepared by the kneading method when there was less intimate interaction between CC and PVP, increasing the concentration of PVP in the solvent led to an increase in its viscosity which increased the thickness of the diffusion layer around the drug particles causing decreasing in the drug dissolution rate [33].

On the other hand, SD systems prepared by the solvent evaporation method using EE100 alone as a polymer exhibited better dissolution in the low pH media. The SE-EE_5_, which consists of CC and EE100 polymer in a 1:5 ratio was able to dissolve 98.5±3.85% of CC in the acidic medium and 100 ± 5.4% of CC dissolved in the acetate buffer during the first 15 minutes of the runs.

Also, the systems SE-EE_3_ and SE-EE_1_ showed an improvement in the dissolution of CC. Still, they were less than that for SE-EE_5_ (SE-EE_5_ > SE-EE_3_ >SE-EE_1_) so by increasing in polymer to drug ratio the dissolution rate increased because the amount of polymer relative to the drug has a significant impact on the dissolution behavior of amorphous SD during dissolution. This improvement in the dissolution of CC in the systems that were prepared using EE100 polymer could be attributed to the fact that EE100 is a pH-sensitive polymer. Since pH-sensitive polymers are polymers that have potential ionizable groups or hydrolyzable compounds dissolved in the acidic medium. By changing the pH of the medium the solubility and the conformation of the pH-sensitive polymers are affected due to the interaction between the solvent molecules and the polymer chains, so in the acidic medium the polymer monomers are induced and the degree of charged monomers is sufficient to induce affinity interaction with the aqueous medium molecules [34].

However, the tertiary SD prepared by using a combination of PVP and EE100 was supposed to increase the solubility in the three-dissolution media. It is worth mentioning that, Prasad *et al*., prepared ternary solid dispersion to enhance the solubility of the weak acidic drug, indomethacin, using a combination of EE100 and PVP K90. Compared with the formula they prepared using a single polymer, they obtained a significant improvement in solubility, which was not the case in this study when PVP K30 and EE100 were combined. Therefore, tertiary SD systems of CC prepared with a combination of PVP and EE100 in the ratio specified in this study had no advantages over SD systems prepared with a single polymer [35].

**Fig 5:**
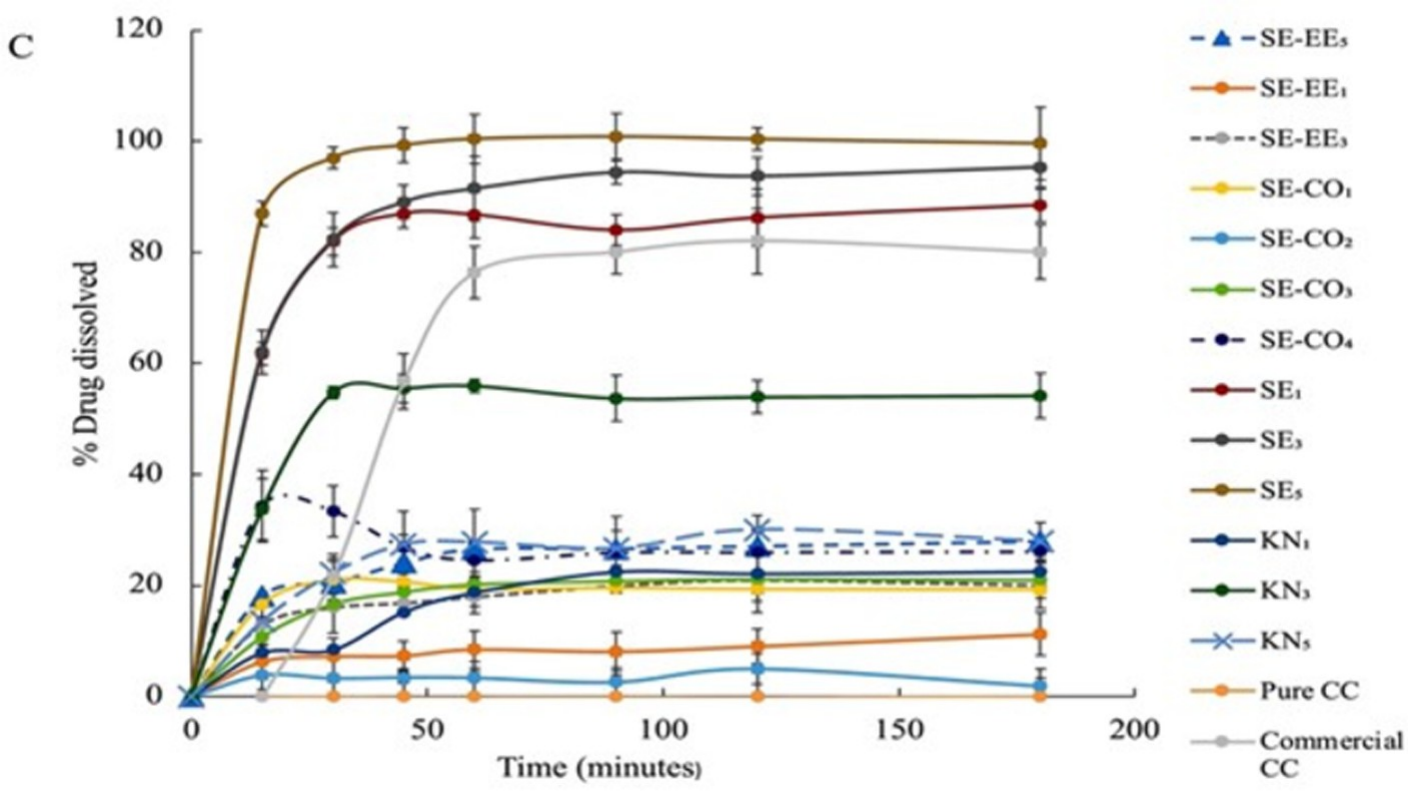
Dissolution profiles of pure CC, commercial CC tablet, and the prepared SDSs in phosphate buffer (pH 6.8).

Moreover, the solubility of KN_1_, KN_3_, and KN_5_ had improved in the basic medium (Fig 5), and increasing the ratio of polymer to drug in the kneading method did not necessarily increase the dissolution rate. The systems SE_1_, SE_3_, and SE_5_ exhibited a higher improvement in the dissolution rate with the dissolution rate increase with increasing the polymer-to-drug ratio in the SE5 system (*p*< 0.001) which could be due to more consistent distribution of the drug within polymer particles in the solvent evaporation method. For the systems SE-EE_1_, SE-EE_3_, and SE-EE_5_ a significant improvement in the dissolution rate was noticed (*p*< 0.05) which only could be due to the conversion of CC into its amorphous form. For the tertiary systems SE-CO_1_-SE-CO_4_, the improvement in the dissolution rate was significant but still less than that of the systems prepared by PVP alone.

As CC’s low bioavailability was attributed to both insolubility and efflux mechanism by the P-gp receptors, thus enhancing the solubility of CC in the simulated gastric and duodenum medium could enhance its bioavailability because it presents the drug in its soluble form in the upper part of the GIT where drug’s absorption mainly takes place, according to a study done by Mai *et al*. who examined P-gp distribution in the GIT, and stated that an increase in the P-gp content along the small intestine from the jejunum to the ileum was shown in male and female humans (jejunum < ileum) and in rats (duodenum < jejunum < ileum). The lower P-gp level and related efflux in the proximal (in contrast to the distal) small intestine explain why the proximal region is the ideal absorption site for drugs that are P-gp substrates [36]. Also, Kagan L. *et al*., concluded that P-gp mediated efflux was a major factor responsible for GI region-specific absorption of talinolol they noticed that the bioavailability of talinolol in humans was markedly decreased when talinolol was perfused in the distal small intestine in comparison to a more proximal location of talinolol and when rectal capsules were administered the bioavailability is reduced by 80% compared with that of an orally administered instant release formulation. To increase the bioavailability of talinolol they suggested two approaches the first was the administration of P-gp substrate drugs with compounds that have P-gp inhibitory properties but the disadvantages were that these inhibitors might increase the potential for adverse effects. The second approach was continuous delivery of the drug to the upper part of GIT which can be accomplished by gastroretentive dosage forms so the drug might be absorbed in the upper part of GIT before reaching the lower intestinal part that is rich with P-gp receptors [37]. Also, Wang Y. *et al*. prepared a solid self-microemulsifying drug delivery system of tacrolimus and incorporate it into a gastroretentive tablet. They stated that their prepared formula enhanced the dissolution and the bioavailability of tacrolimus by releasing the drug in the upper part of GIT so the drug is to be absorbed in the proximal P-gp poor region of the intestine rather than the far and P-gp rich region [38].

Furthermore, improving the solubility of BCS II drugs which are of low solubility, high permeability, and substrates for P-gp receptor-like CC in the gastric medium would make them suitable for gastroretentive drug formulations [39], thereby increasing their bioavailability and therapeutic activity. The SE-EE_5_ system was selected for further investigations and the mechanism involved in enhancing the solubility of CC was explored.

### 3.6. Simulated pharmacokinetic IVIVC study

For a drug to be absorbed into the bloodstream to reach its site of action it should be present in a solution form in the GIT at the absorption site [2]. *In vitro*, dissolution testing is conducted to estimate or predict the dissolution of the drug in the GIT or *in vivo*. Therefore, some form of relationship between these two dissolution types is desirable and it is commonly referred to in the literature as *in vitro*-*in vivo* correlation (IVIVC) [22]. FDA defines IVIVC as a predictive mathematical model to describe the relationship between an *in vitro* property (usually the extent or rate of drug release), and a relevant *in vivo* response e.g., plasma concentration or amount of drug absorbed [40].

Developing and conducting a dissolution test based on such a relationship not only enhances the reliability of an *in vitro* test but also provides several benefits such as a reduction of the required number of *in vivo* studies in humans, thus simplifying the development and modification of the drug products and lowers overall cost.

In the case of oral dosage forms, as highlighted by the FDA, it is always possible to correlate *in vitro* and *in vivo* data for formulations where the absorption of the API is limited by the dissolution rate (the dissolution of the drug is the rate-limiting step). Hence, IVIVC works better with class II BCS drugs. Since CC is a class II drug with low solubility and high permeability, IVIVC could give a clear picture of the pharmacokinetics parameters of CC.

**Fig 6:**
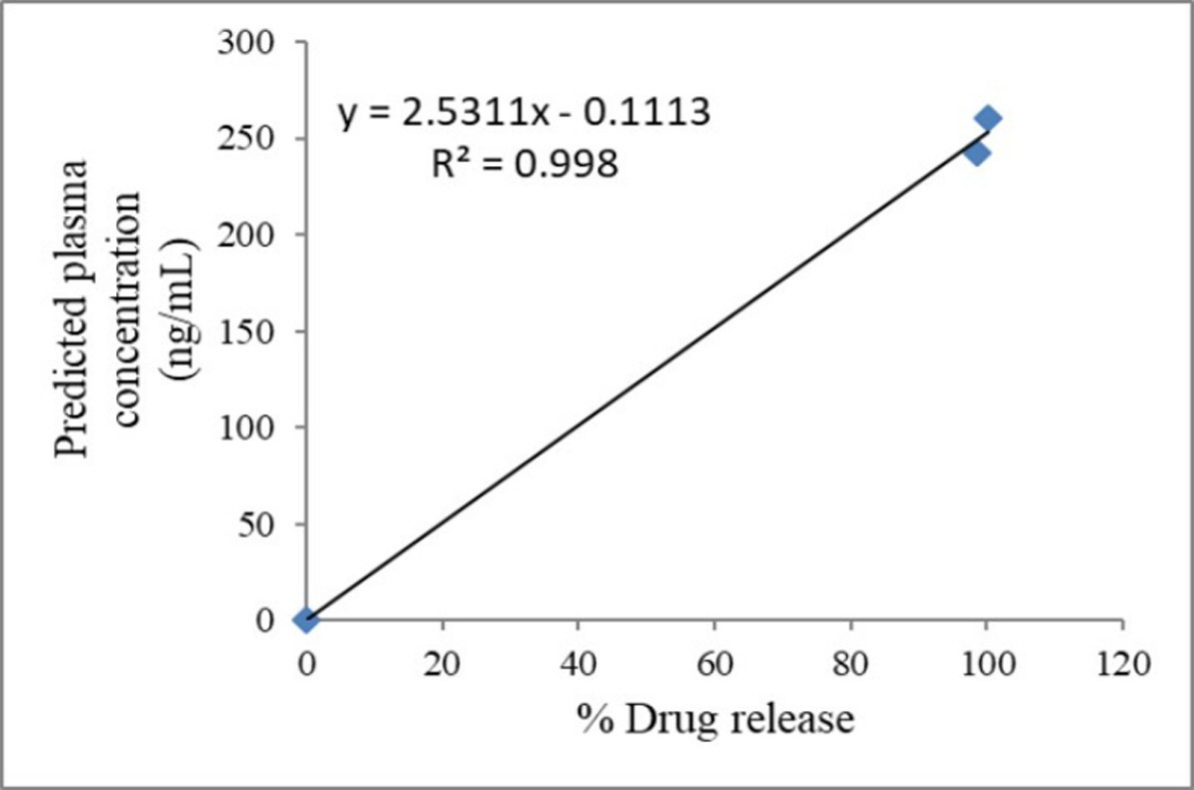
The IVIVC–*In vitro* drug dissolution versus plasma concentration of CC loaded in SE-EE_5_.

**Fig 7:**
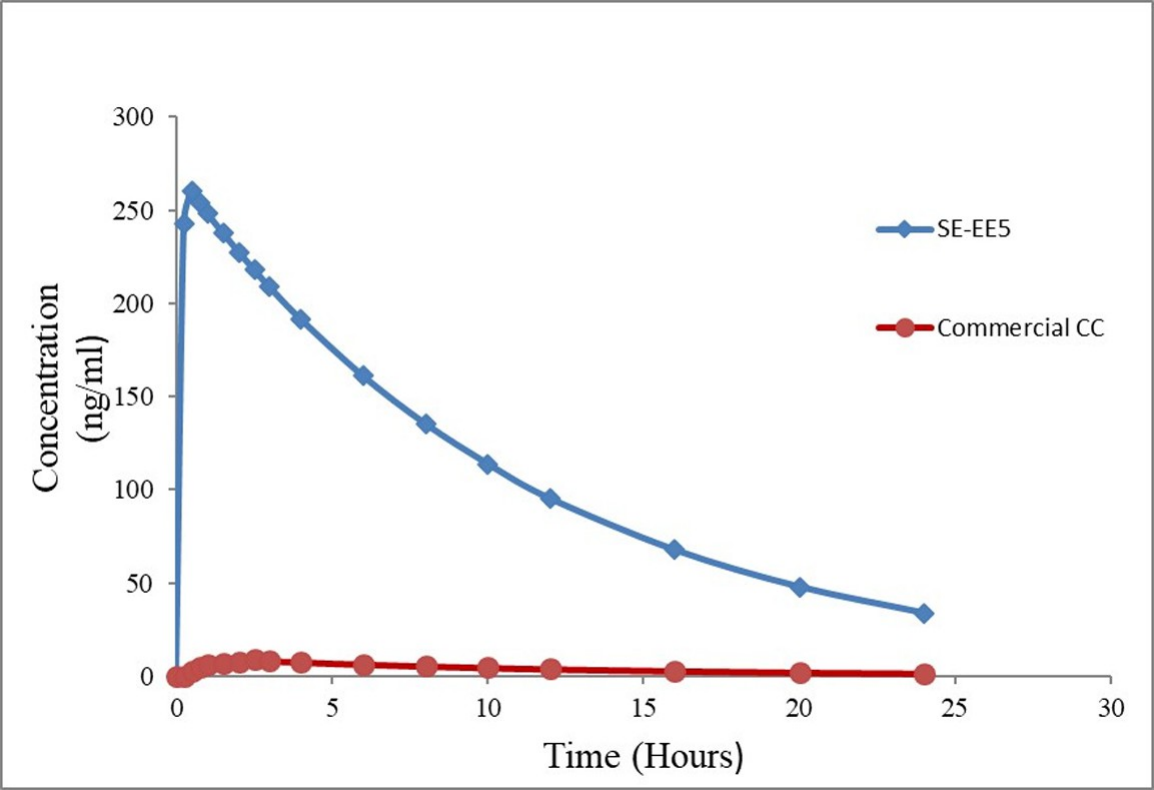
Predicted plasma profile for the commercial candesartan cilexetil tablet and SE-EE_5_.

There are two methods of establishing IVIVC, the one-stage convoluted method, and the two-stage deconvoluted method. The convolution approach uses the *in vitro* dissolution data and pharmacokinetic characteristics of the drug to obtain plasma drug concentration. On the other hand, the deconvolution method is a two-stage procedure, which involves the development of formulations with different release rates and their dissolution testing, providing *in vivo* plasma concentrations for the formulations, and finding *in vivo* absorption or dissolution time profiles. The results of IVIVC showed that the system SE-EE_5_ has a higher predicted AUC and C_max_ (Table 6) than that for the commercial tablet, with a linear correlation coefficient (R^2^ of 0.998) (Fig 6). The predicted plasma profile for the commercial tablet and SE-EE_5_ is illustrated in Fig 7. These results indicated that enhancing the dissolution of CC thousands of times by formulating it as a solid dispersion with polymer EE100 could drastically enhance the bioavailability of class II drug CC.

**Table 6:** Predicted pharmacokinetic parameters of SE-EE_5_.

Predicted PK parameters	Commercial CC tablet	SE-EE_5_
AUC ng * hr/mL	113.07	3094
C_max_ ng/mL	8.85	259.95
T_max_ (hr)	2.5	0.5

### 3.7. DSC study

**Fig 8:**
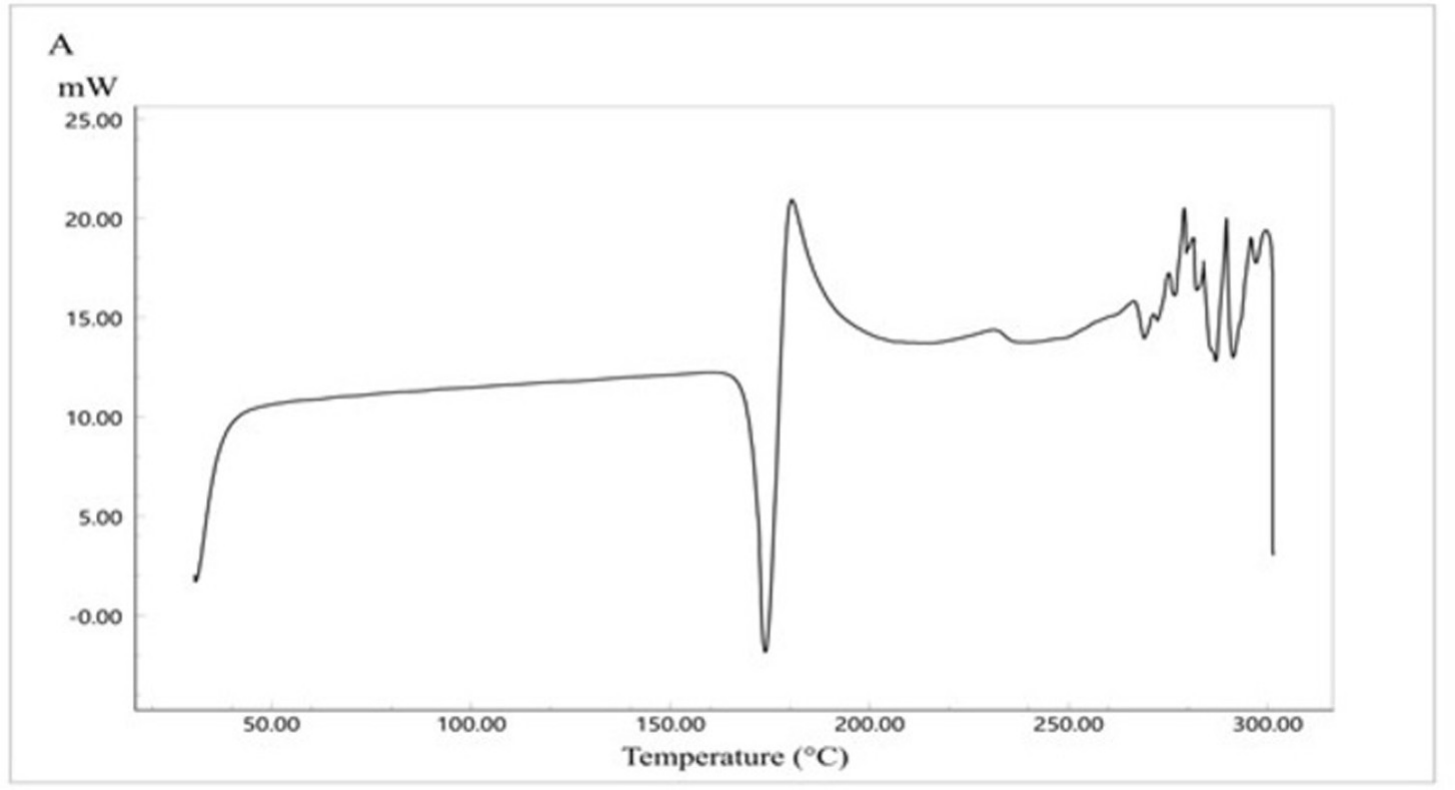
DSC thermogram of CC pure drug.

**Fig 9:**
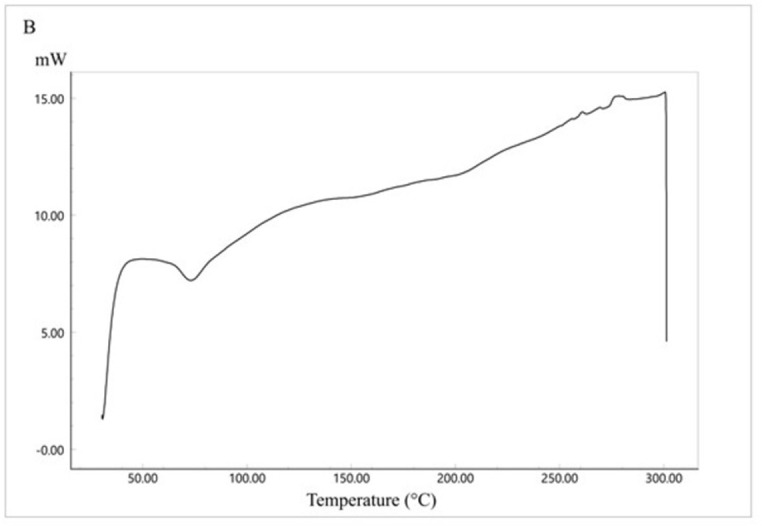
DSC thermogram of EE100 polymer.

**Fig 10:**
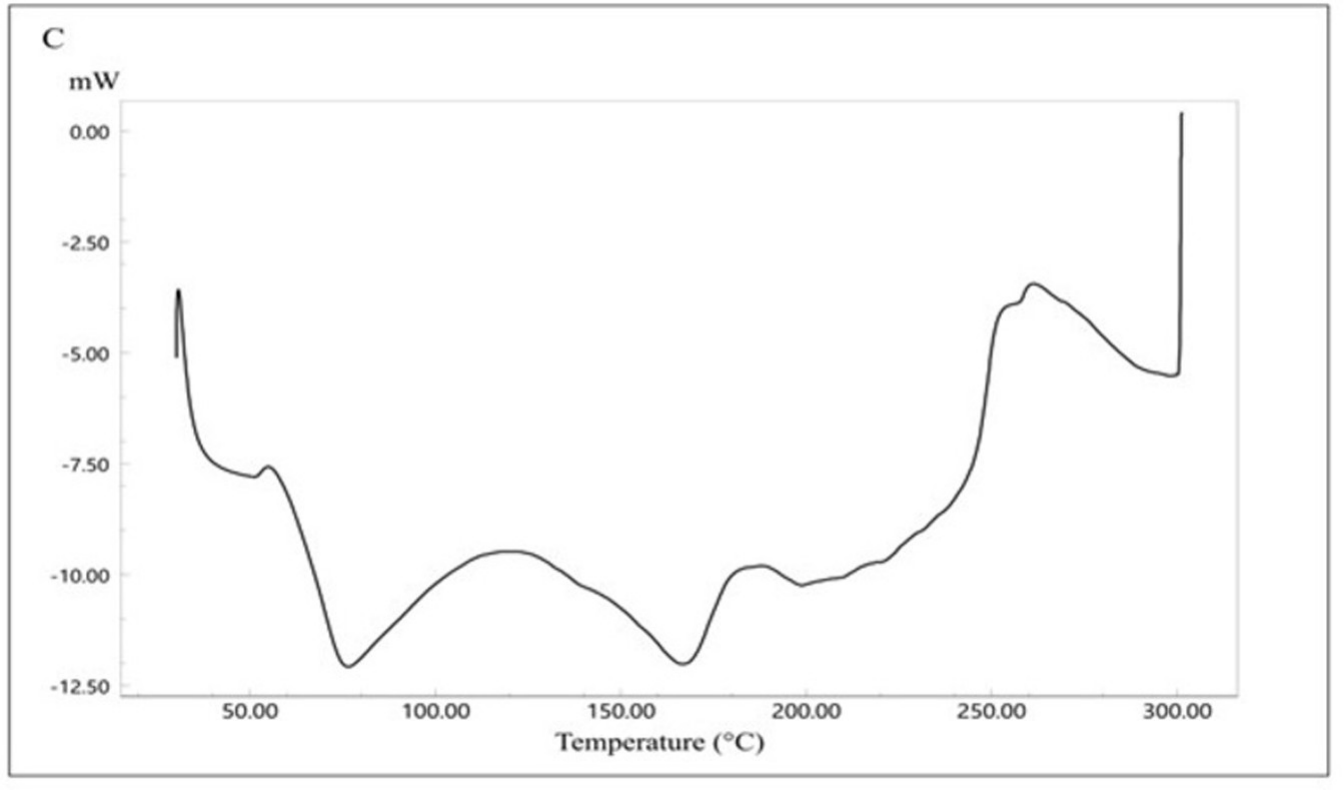
DSC thermogram of CC-EE100 physical mixture.

**Fig 11:**
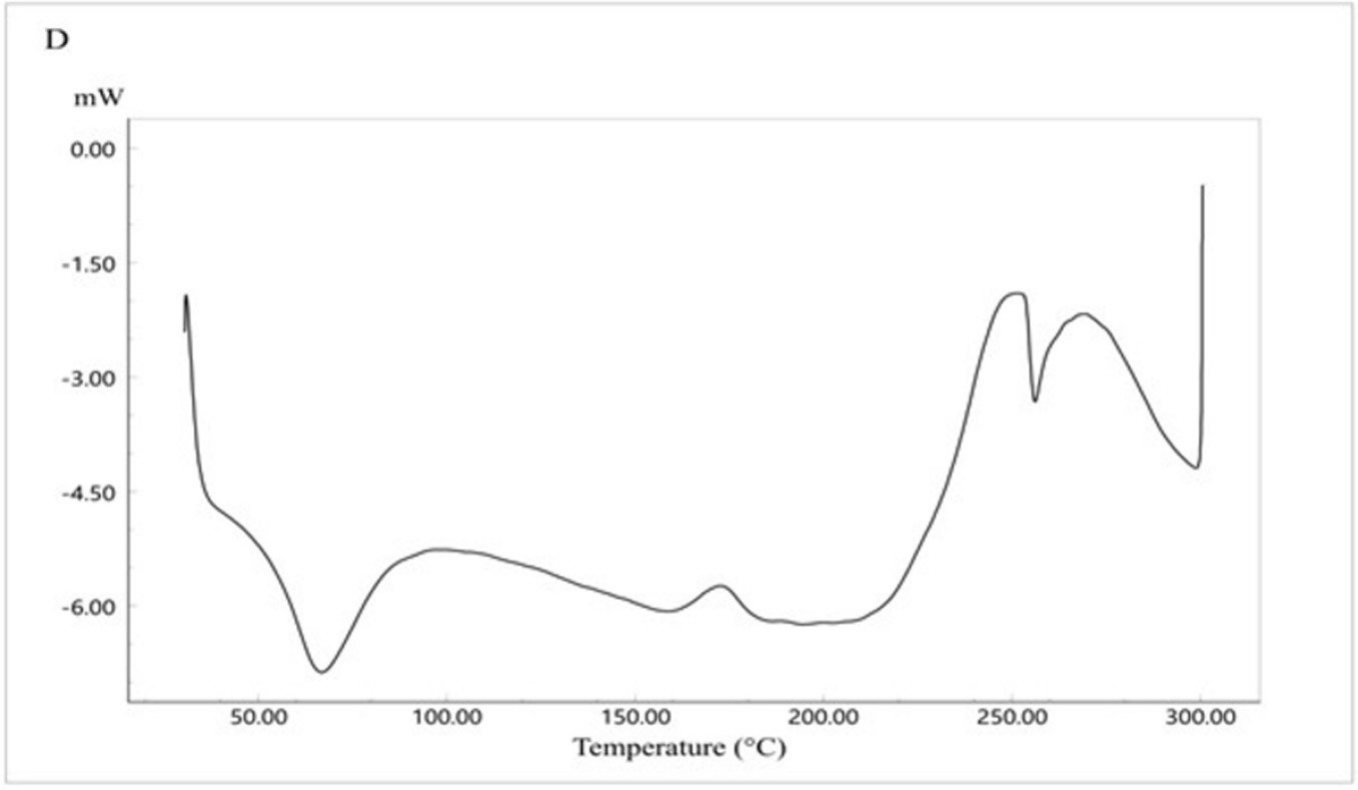
DSC thermogram of the optimized formulation SE-EE_5_.

The thermal analysis of CC is presented in Fig 8). CC shows a sharp endothermic peak at 173.77°C representing the drug melting point [41]. The endothermic peak is followed directly by an exothermic peak which could correspond to polymorphic transformation [42]. Fig 9 represents the DSC thermogram of Eudragit E100. Eudragit, as a polymer, has a glass transition temperature of 50°C. The physical mixture of the formula components (Fig 10) showed the polymer glass transition temperature and the CC melting point with a change in intensity which could be due to the solubilization of the drug by the fused polymer during heating. For the optimized SD system (Fig 11), the melting point for CC disappeared, indicating the conversion of the crystalline drug into an amorphous form, explaining the excellent solubility of the formula compared with the pure drug. This conversion is a result of the molecular dispersion and incorporation of the API within the polymer matrix. However, the DSC thermogram of the SE-EE_5_ showed an endothermic peak at about 256.16°C, which could be occurred due to the fusion decomposition of the components [43].

### 3.8. XRD study

**Fig 12:**
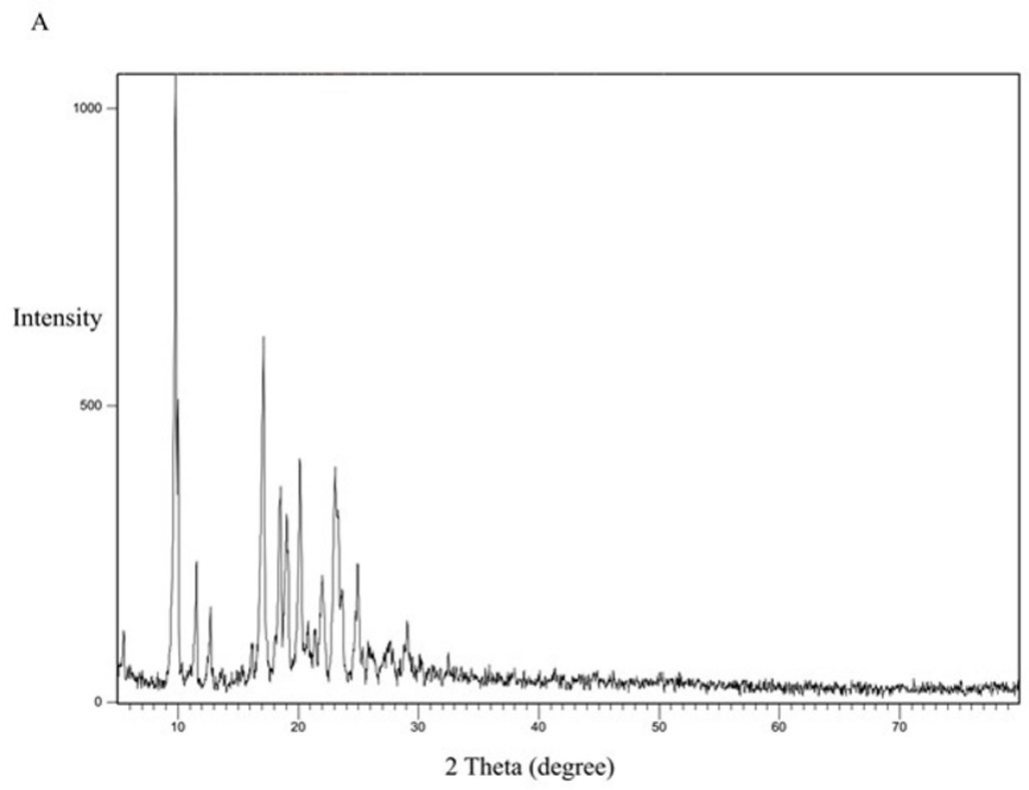
XRD spectra of CC pure powder.

The XRD study was performed to study the crystallinity of the drug and the polymorphic changes in the API. The XRD study for the pure drug is shown in Fig 12. It shows 2θ values at 9.8°, 17.2°, 18.5°, 19°, 20.3, and 23.2°. The characteristic peak recorded at 9.8° indicates that CC is present in a crystalline polymorph I [42].

**Fig 13:**
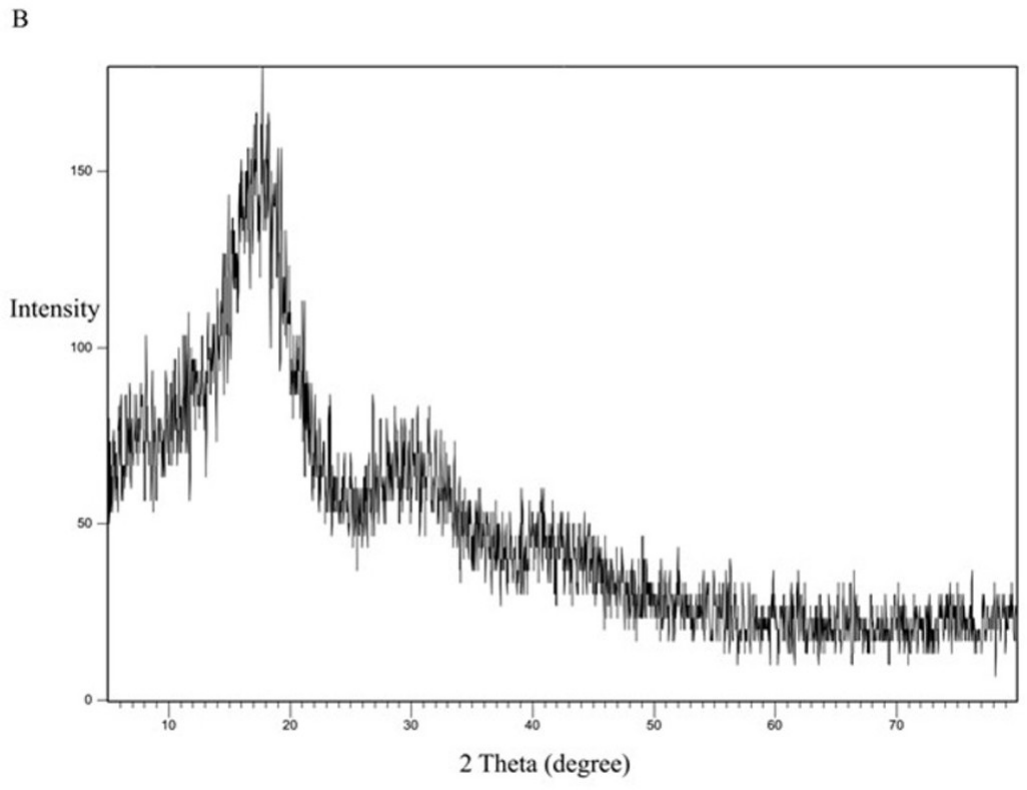
XRD spectra of EE100 polymer.

**Fig 14:**
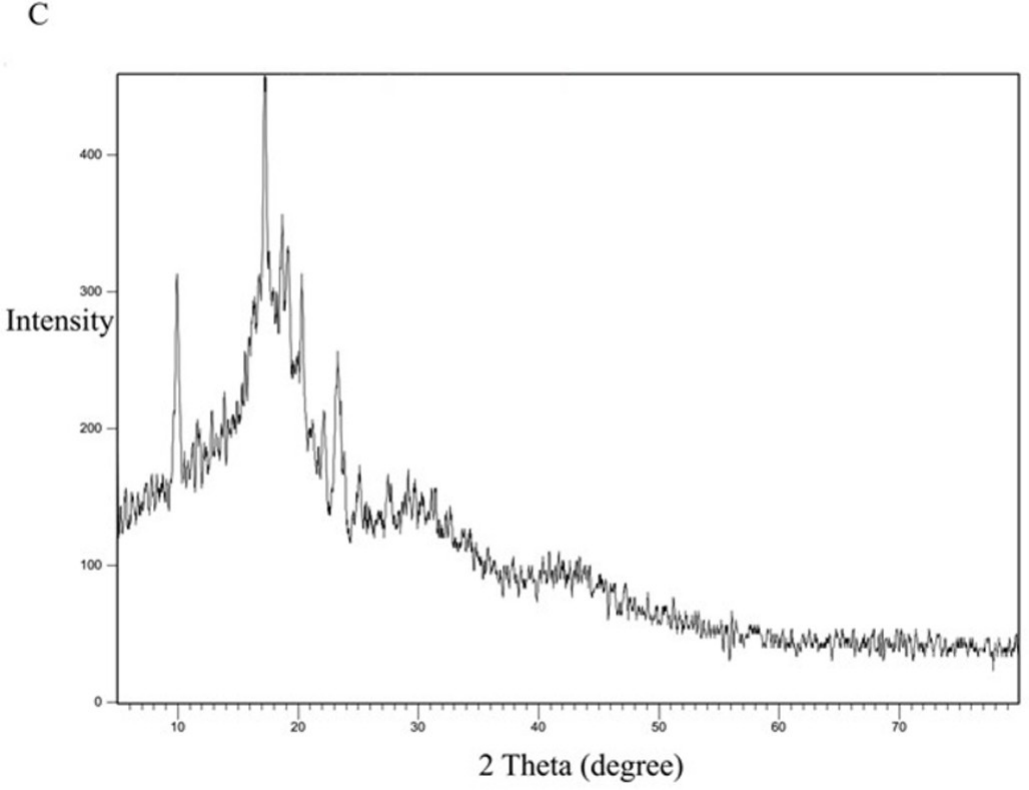
XRD spectra of CC-EE100 physical mixture.

**Fig 15:**
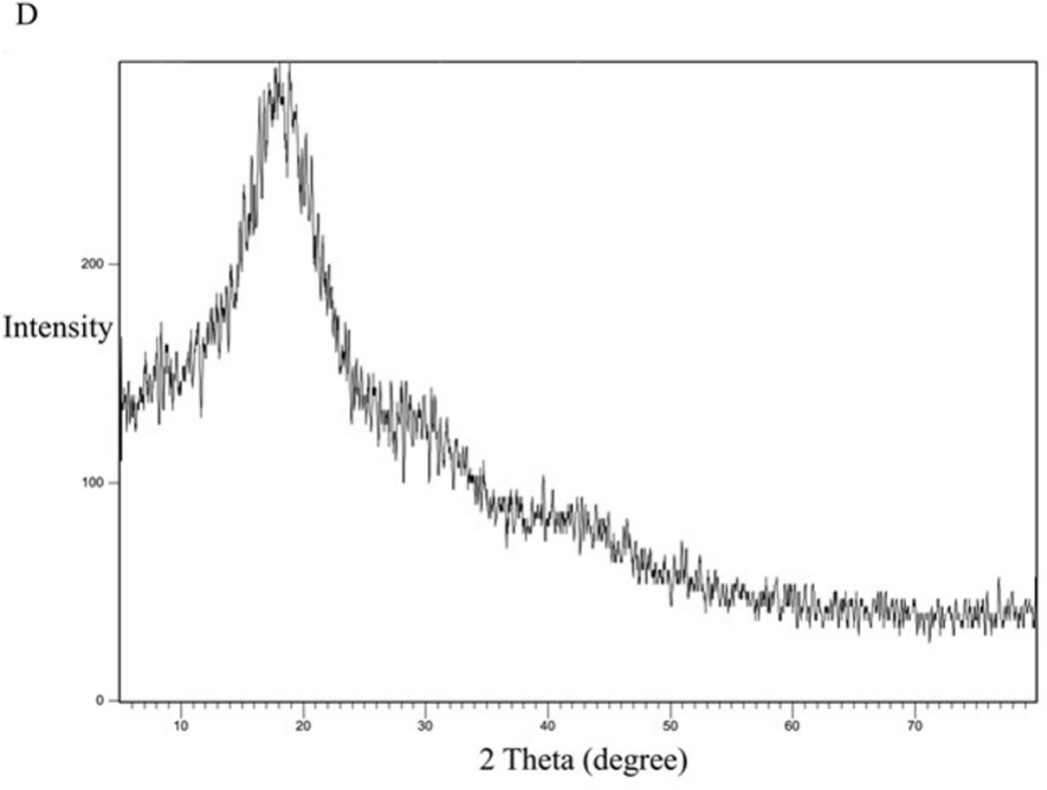
XRD spectra of the optimized formulation SE-EE_5_.

Fig 13 represents the XRD study of Eudragit E100 alone. The XRD pattern of Eudragit E100 exhibited no significant diffraction peaks, which is in line with the amorphous nature of this acrylic polymer. The DSC curve of the physical mixture of the polymer and the drug (Fig 14) represents the characteristic peaks of CC, confirming that CC is in its crystalline form. However, in the XRD of the selected solid dispersion system SE-EE_5_ represented in Fig 15, the peaks disappeared, indicating the conversion of the crystalline CC to an amorphous form, explaining the extreme improvement in solubility.

**Fig 16:**
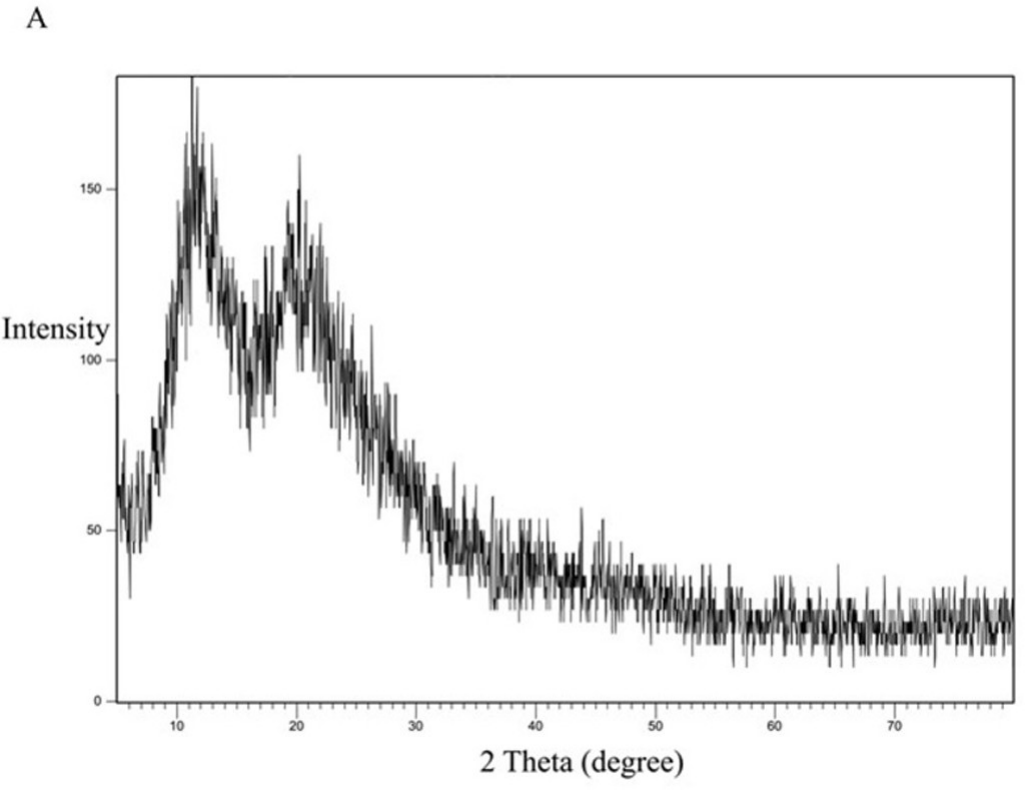
XRD spectra of PVP polymer.

**Fig 17:**
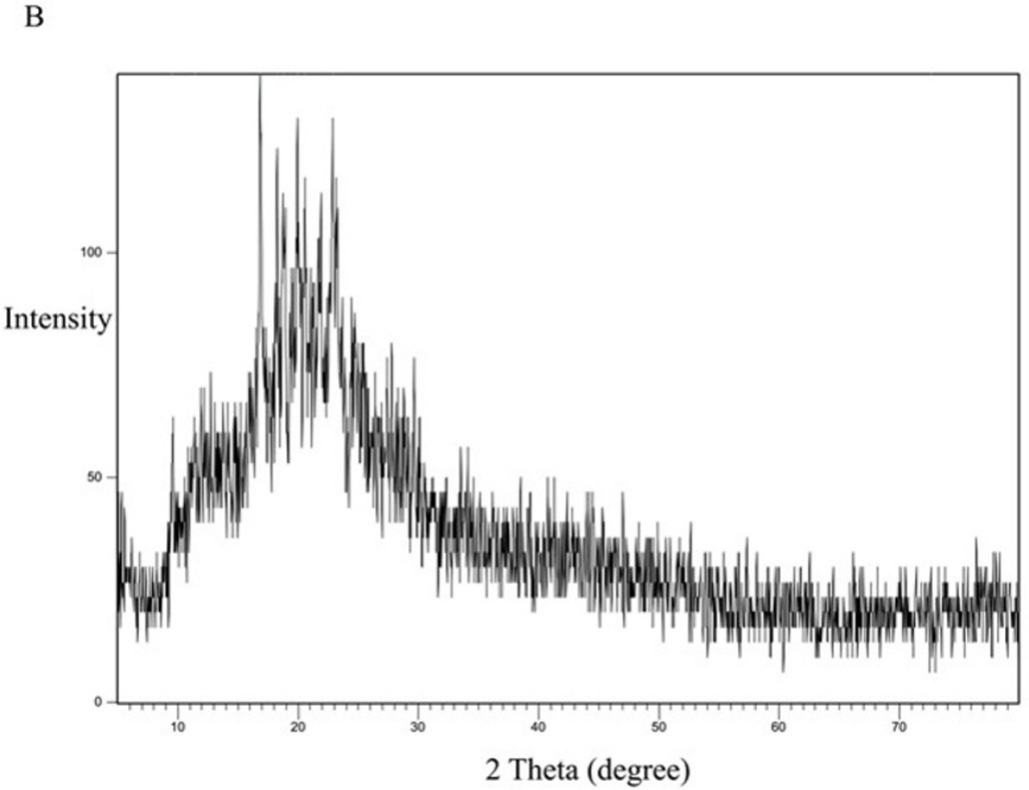
XRD spectra of CC-PVP physical mixture.

**Fig 18:**
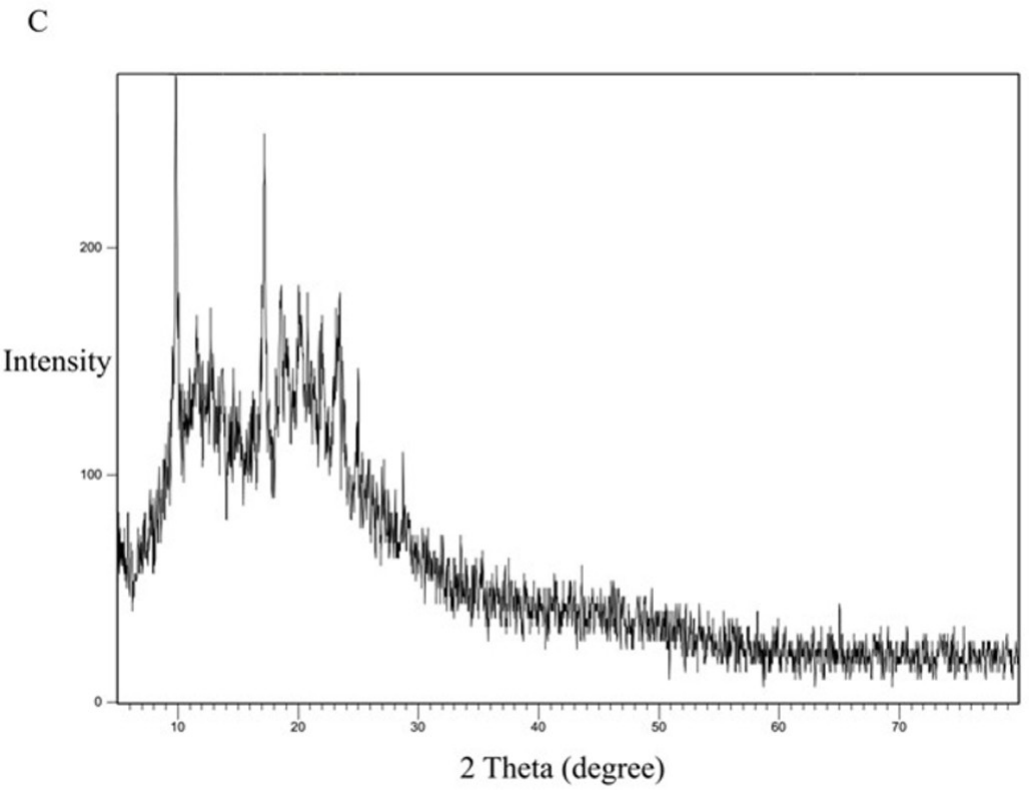
XRD spectra of the solid dispersion KN_3_.

**Fig 19:**
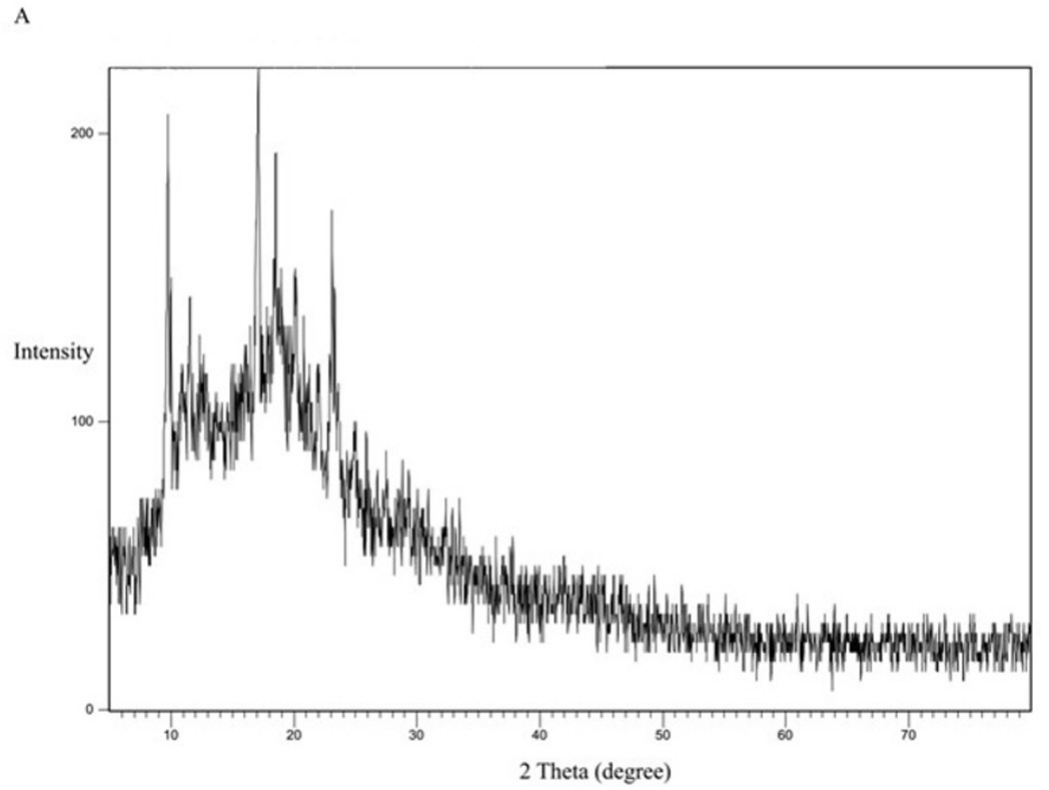
XRD spectra of SE-CO_3_ physical mixture.

**Fig 20:**
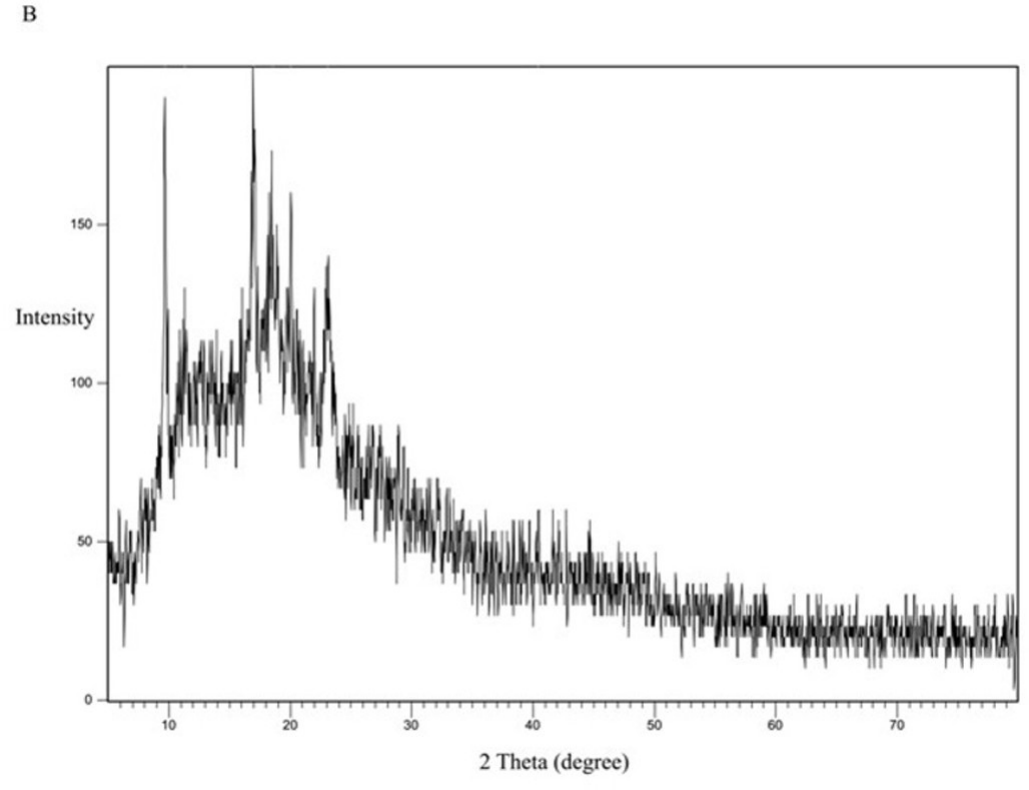
XRD spectra of the solid dispersion SE-CO_3_.

For comparison, the formula KN_3_ (Fig 16) with a dissolution profile less than that of the SE-EE_5_ was examined for crystallinity, with the polymer PVP (Fig 17) and the physical mixture of CC and PVP (Fig 18). PVP showed no characteristic peaks, while the physical mixture and the SD system KN_3_ showed the same characteristic peaks of CC, indicating that not all the CC was converted into an amorphous form explaining the low improvement in solubility and exhibited by the system and indicating that for CC, the conversion into an amorphous form is one of the main mechanisms required to improve the solubility. Moreover, neither the physical mixture nor the system SE-CO_3_ showed that all the CC is converted into an amorphous form (Figs 19 and 20).

The XRD results were consistent with the dissolution study, as SDSs that gave a better dissolution profile showed the disappearance of the characteristic peaks of the API, declaring the change of API into an amorphous form [44].

### 3.9. FT-IR study

The FT-IR spectrum of the system usually contains peaks that are shifted relative to those found in the spectrum of the physical mixture of drug and polymer. This is due to specific interactions between the functional groups of the drug and polymer, usually hydrogen bonding but it also could be an ionic or hydrophobic interaction. If these interactions differ in strength or extent relative to the physical mixture in these systems, changes in FT-IR peak position and shape occur for the functional groups involved [45].

**Fig 21:**
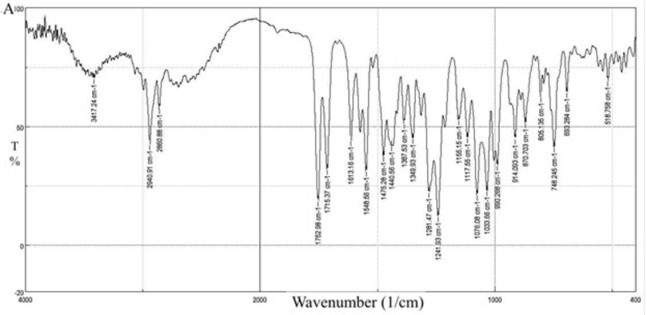
FT-IR spectra of CC pure drug.

The spectrum of CC is shown in Fig 21. The spectra showed N-H stretching at 3417.24 cm^-1^ stretching aliphatic and aromatic C-H band at 2860.88, 2940.91, and 3000 cm^-1^; carbonyl group stretching from the carbonate -OC (= O)O- moiety with an intense band at 1752.98 cm^-1^; an ester carbonyl group at 1715.37 cm^-1^; an N-H bending at 1613.16 cm^-1^; an aromatic C-N stretching at 1349.93 cm^-1^ and C-O ether at 1033.66 cm^-1^. These absorption bands are summarized in Table 7. The spectra showed matching with the IR spectrograph in previous studies [46].

**Table 7:** Characteristic FT-IR peaks of CC and Eudragit E100.

Wavelength (cm^-1^)	Type of the bonds	
	CC	Eudragit E100
3417.24	N-H stretching	
2860.88, 2940.91, and 3000	Aliphatic and aromatic C-H stretching	
1613.16	N-H bending	
1752.98 and 1715.37	C = O stretching	
1349.93	C-N stretching	
1033.66	C-O ether	
2820 and 2770		C-H stretching in Dimethylamino group
1485.88 and 3010.3		C-H stretching
1155.15		Ester group vibration stretching
1732.73		C = O stretching

**Fig 22:**
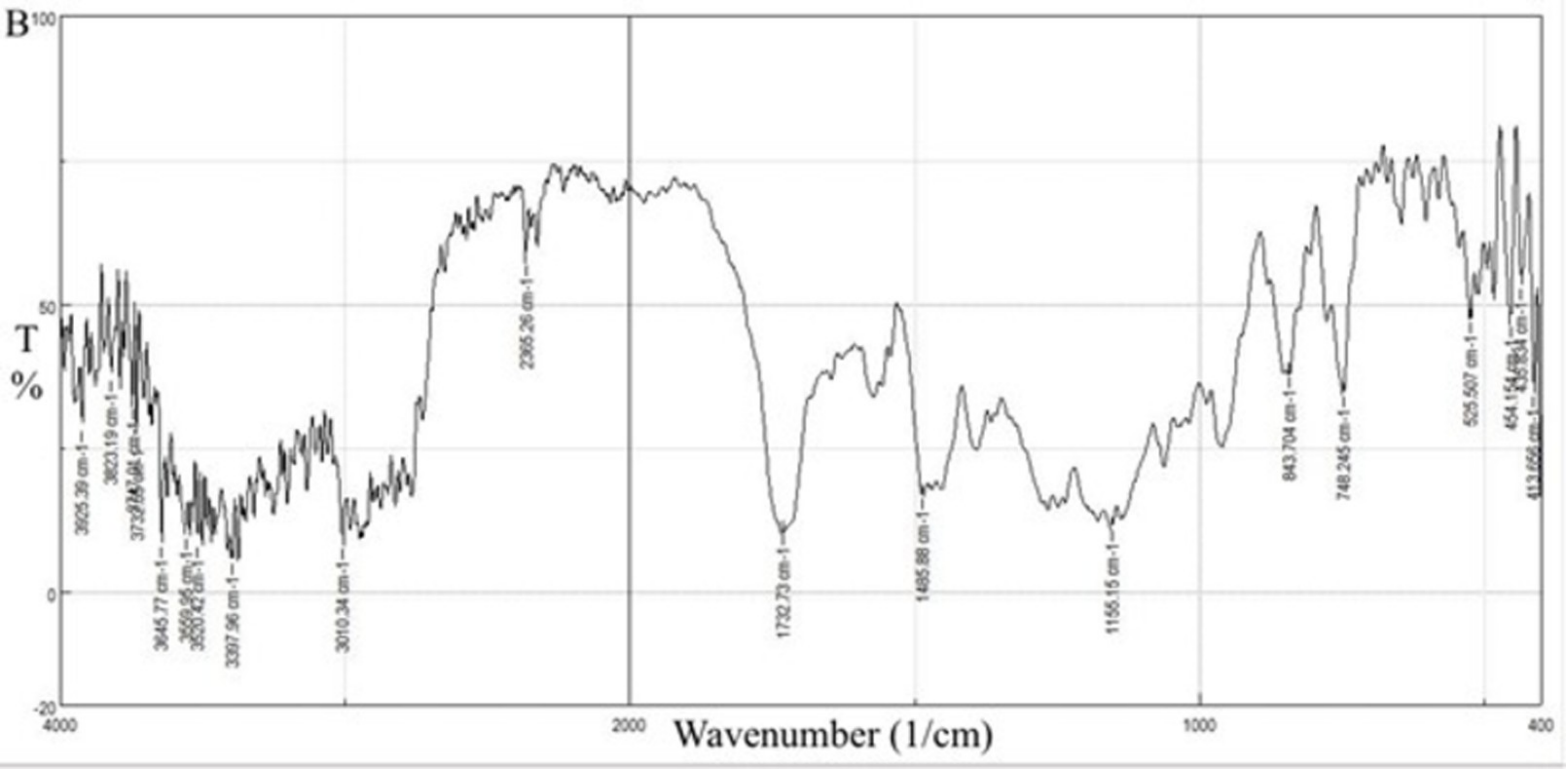
FT-IR spectra of EE100 polymer.

Fig 22 shows the characteristic FT-IR peaks of Eudragit E100. The C = O ester vibration occurs at 1732.73 cm^-1^, and the ester groups at 1155.15 and 1250. In addition, C-H vibration could be seen at 1485.88 and 3010.34 cm^-1^. The absorptions at 2770 and 2820 cm^-1^ can be assigned to the dimethylamino groups [13]. These peaks are summarized in Table 7.

**Fig 23:**
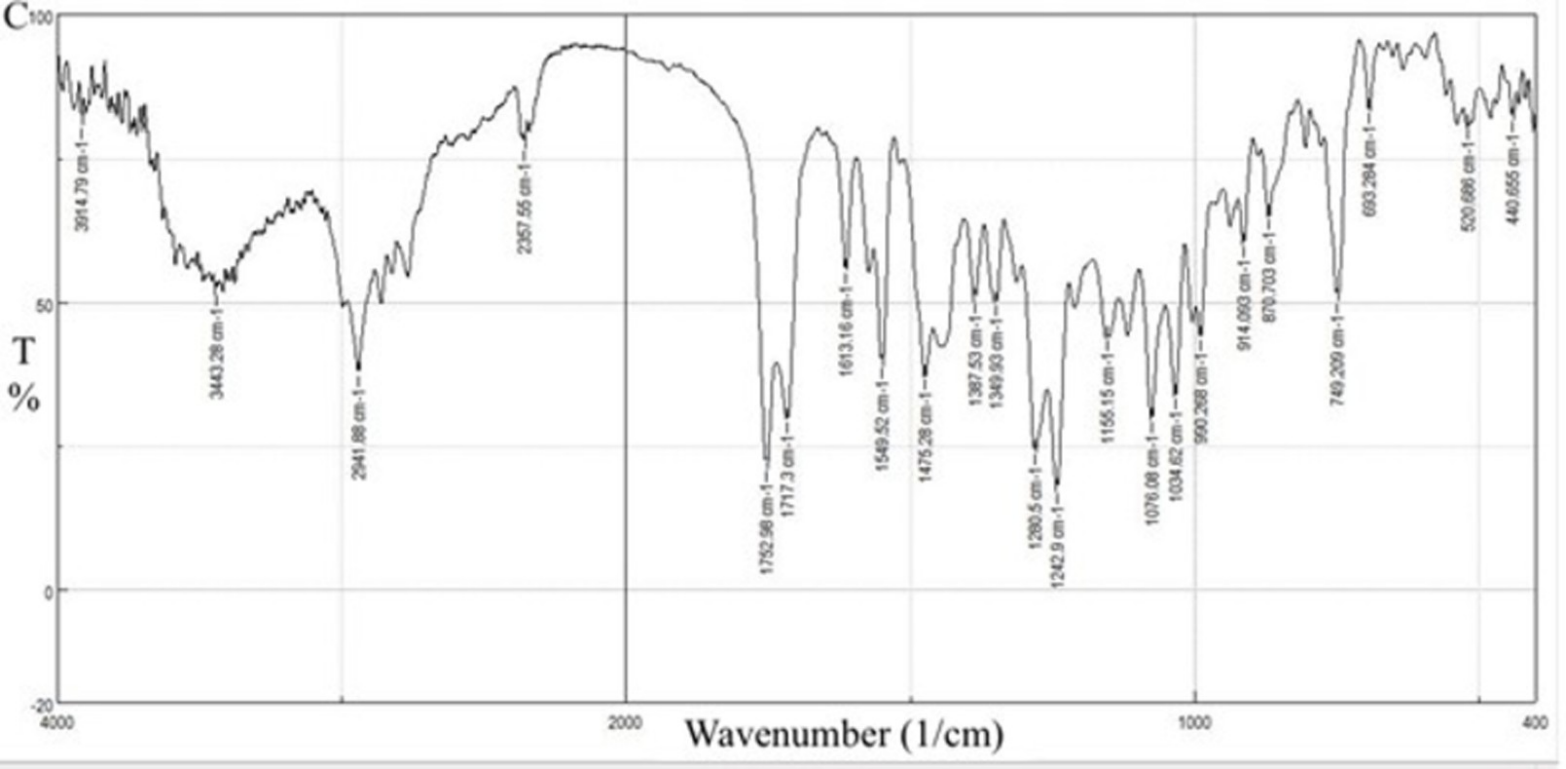
FT-IR spectra of CC-EE100 physical mixture.

Fig 23 represents the FT-IR results for the physical mixture and shows the same characteristic peaks for CC and EE100, no extra peaks are reported peaks indicating no chemical interaction.

**Fig 24:**
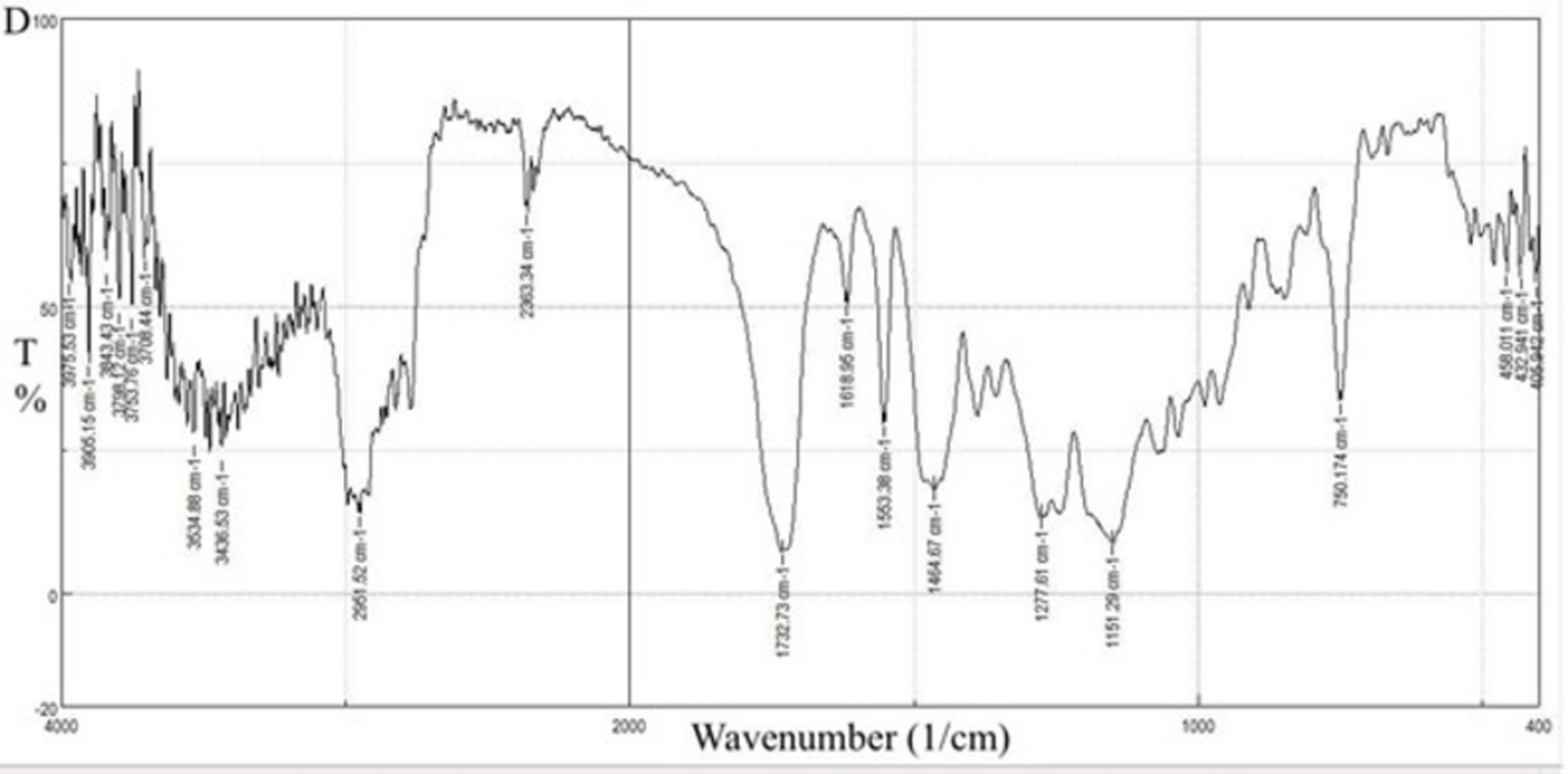
FT-IR spectra of the optimized formulation SE-EE5.

For the selected solid dispersion system in Fig 24, the characteristics of C = O peaks of CC at 1715.37 cm^-1^ and 1752.98 cm^-1^ changed in position and superimposed with the C = O peak of EE100. The peaks at 3417.24 cm^-1^ and 1613.16 cm^-1^ belonging to the N-H stretching and vibration of CC respectively changed in positions and intensities. The peaks of EE100 at 2820 cm^-1^ and 2770 cm^-1^ belonging to the C-H stretching of the methyl group in the dimethyl amino group also change in position and intensity. All these changes in peak position and intensities indicate the formation of hydrogen bonds assisting in explaining the improved dissolution profile [46].

### 3.10. SEM examination

**Fig 25:**
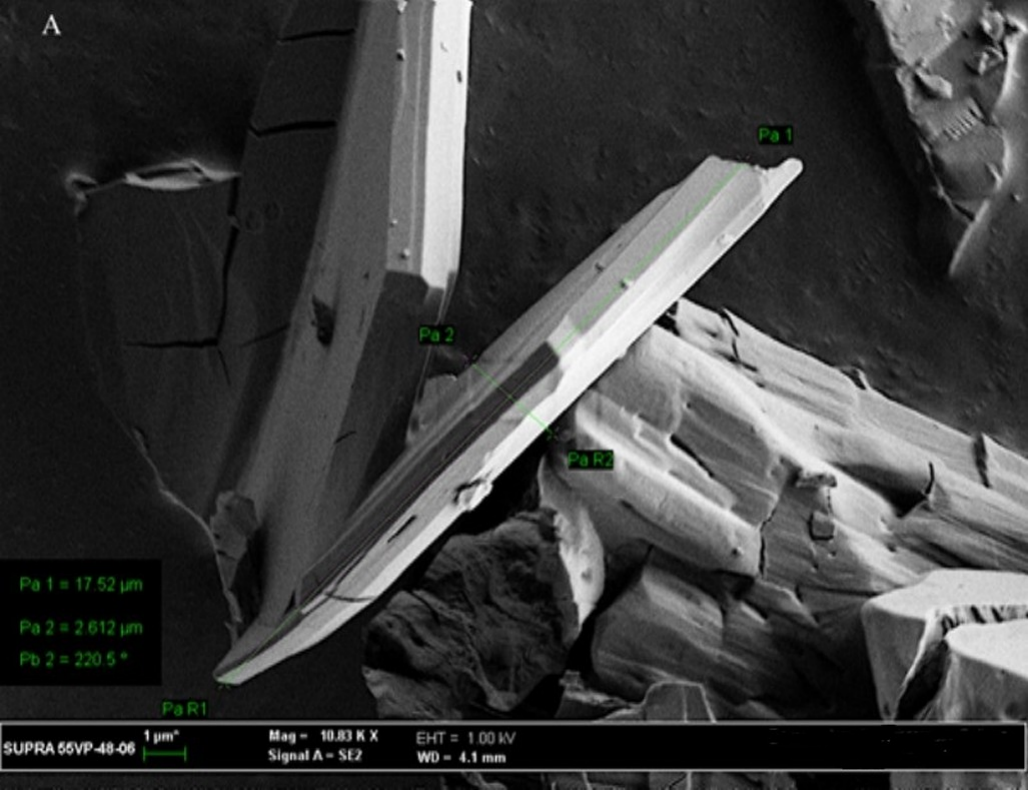
SEM photographs of CC pure drug.

**Fig 26:**
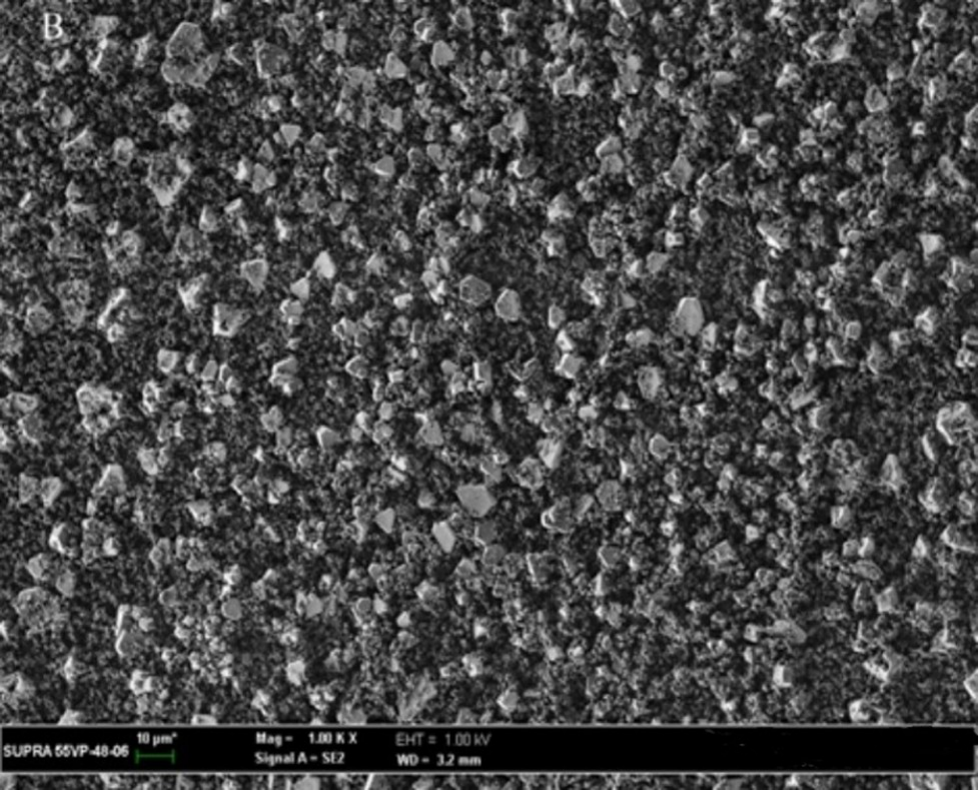
SEM photographs of CC-EE100 physical mixture.

**Fig 27:**
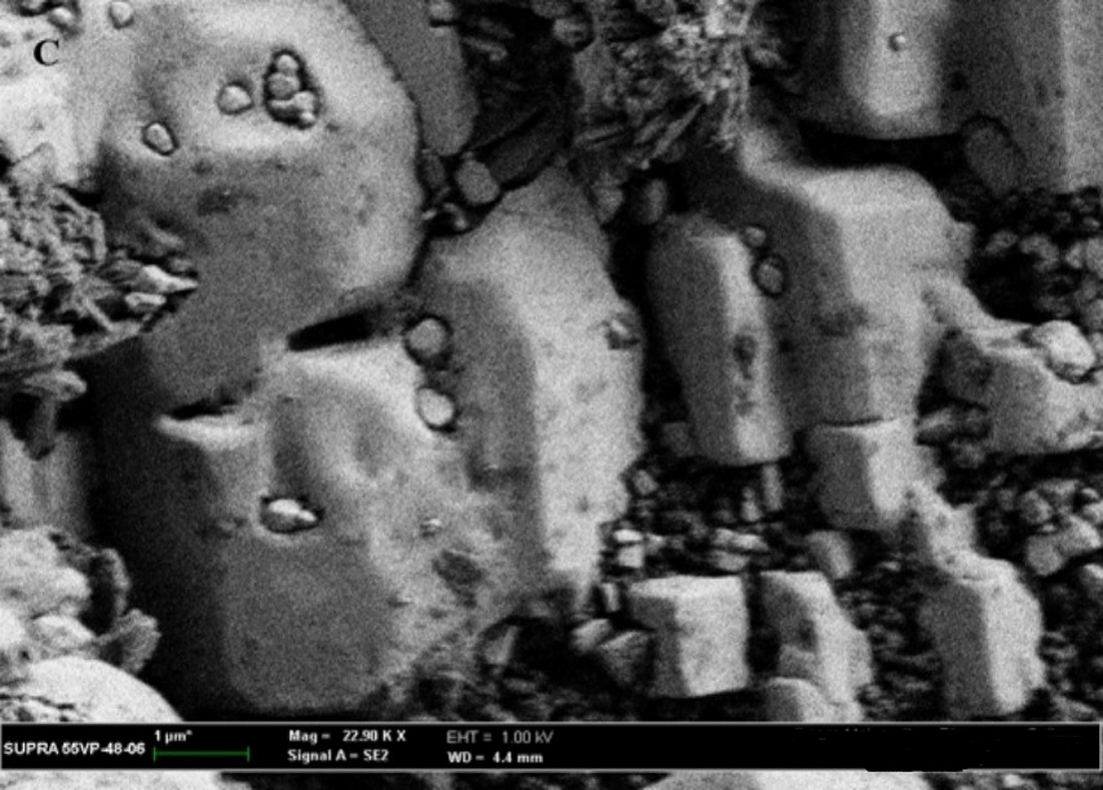
SEM photographs of the optimized formulation SE-EE_5_.

The SEM micrographs of the CC powder showed a regular-shaped crystalline particle (Fig 25). Fig 26 represents the CC- EE100 physical mixture which reflects the crystal nature of CC and the amorphous nature of EE100. However, the disappearance of the crystalline structure in Fig 27 which represents the SEM of SE-EE_5_ indicates the conversion of the drug crystal into an amorphous form. The SEM study came in consistence with the dissolution, the XRD, the DSC, and the FTIR results and ascertained the conversion of CC crystals into an amorphous form.

### 3.11. DLS analysis

Two milliliters of 2 mg/mL SE-EE_5_ solution in HCl were poured into a disposable cuvette and tested by a NanoZS Zeta sizer. The results of the zeta sizer revealed that the average diameter of the nanoparticles was 203.2±0.418 nm and uniform particle size distribution (PDI of 0.189). These results indicated the formation of SE-EE_5_ nanoparticles [47].

### 3.12. STEM study

**Fig 28:**
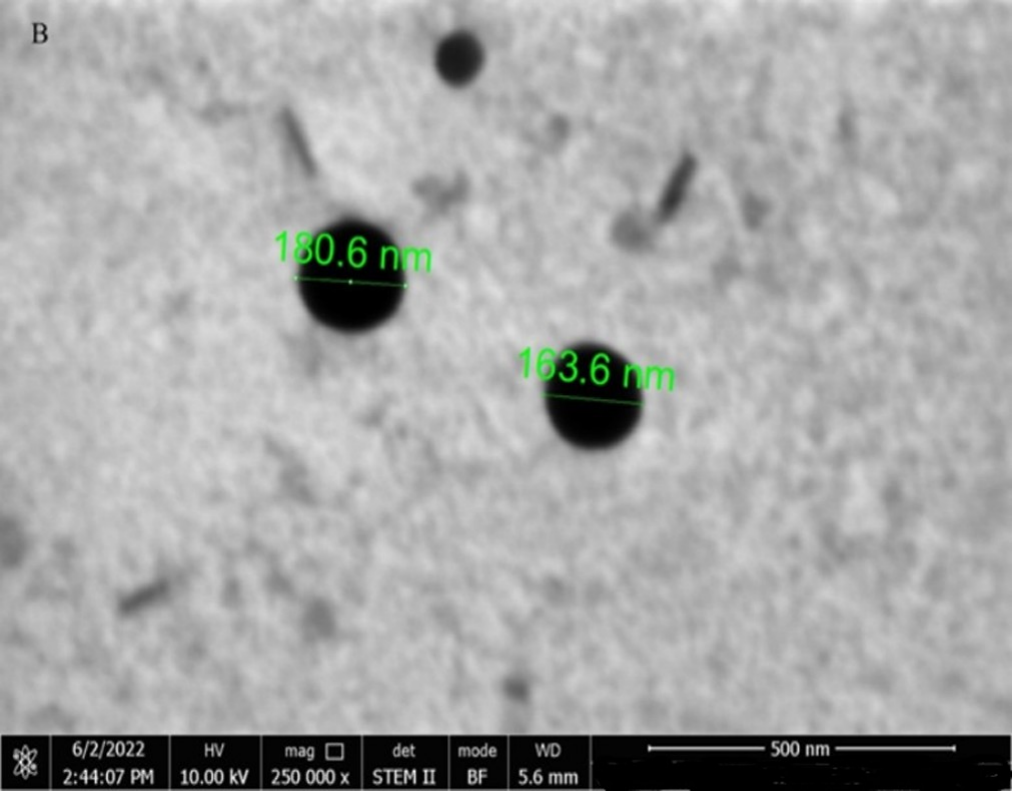
STEM photographs showing the spherical nanoparticles formed after dissolving the SE-EE_5_.

The solution of SE-EE_5_ in HCl in a concentration of 2 mg/mL was spread on a copper grid previously covered with a carbon layer. The STEM result confirmed the formation of nanoparticles which were observed directly as black spherical shapes as illustrated in Fig 28.

The results of DLS and STEM confirm the formation of nanoparticles which adds to understanding one of the mechanisms that enhanced the dissolution of CC when prepared as solid dispersion with the polymer EE100. The amphiphilic nature of EE100 makes it able to form nanoparticles upon dissolution in acidic media as was mentioned by Lin X *et al*. when they proved that dimethyl aminoethyl methacrylate polymer could reveal self-assemble performance in acidic media and could be formed into nanoparticles to enhance the solubility of poorly water-soluble drugs [48].

Combining the results of all the tests done on the SD system SE-EE_5_, the extremely improved solubility of CC could be attributed to both the molecularly dispersed drug particles within the polymer matrix, the conversion of the crystalline nature of the CC to the amorphous form, the formation of nanoparticles during dissolution due to the amphiphilic nature of the EE100 polymer, and the ionizable nature of EE100 polymer.

### 3.13. Stability study

The SE-EE_5_ powder showed no aggregation or change in color or texture at the end of the storage duration. The FT-IR spectrum presented the same peaks with no change after 12 months. The XRD study also confirmed the absence of the CC characteristic peaks indicating that the SDs still in the amorphous. In addition, the HPLC chromatogram showed the same peak and area for the SE-EE_5_ solid powder before storage, and the drug content was 97.2 ±0.04%. Finally, no significant changes in the dissolution profile were observed. The results demonstrated the effectiveness of the optimized solid dispersion system SE-EE_5_ in stabilizing the amorphous state of CC. The stabilization of the amorphous state is crucial because amorphous drugs tend to revert to their more stable crystalline forms over time, leading to decreased solubility and potential loss of therapeutic efficacy. Through the solid dispersion technique, the study sought to overcome this challenge by dispersing CC molecules within a carrier matrix, preventing their re-crystallization and maintaining the amorphous state for an extended period [49].

The FT-IR spectrum, XRD pattern, and dissolution profile in HCl and phosphate buffer of SE-EE_5_ after 12 months of storage are provided in S1 and S2A–S2C Figs.

## 4. Conclusions

Based on the preceding findings, it is evident that the solubility of CC experienced a remarkable enhancement of over 27 thousand times through the application of solvent evaporation method to prepare solid dispersion systems utilizing Eudragit E100 as a carrier polymer at a 1:5 (*w/w*) drug-to-polymer ratio. The IVIVC study corroborated that improving CC solubility could significantly augment its bioavailability, particularly crucial for CC, classified as class II according to the BCS, where dissolution represents the limiting factor in drug bioavailability. Both XRD and FTIR analyses demonstrated the ability of the polymer EE100 to transform the crystalline CC into an amorphous state. Moreover, EE100 solid dispersion led to the formation of nanoparticles further bolstering CC solubility. These findings were supported by DLS and STEM analyses, revealing the presence of nanoparticles with an average diameter of 203.2 nm and confirming the spherical morphology of the nanoparticles, respectively. Accordingly, this study shows that CC’s conversion into an amorphous form combined with EE100’s ionization and formation of nanoparticles contributed to our understanding of solubility enhancement mechanisms. Stability assessments affirmed the robustness of the selected system when stored for 12 months at 25°C. This investigation laid the groundwork for future endeavors aiming to integrate the SE-EE_5_ system into a gastroretentive delivery platform and conduct *in vivo* tests to ascertain whether the increased dissolution of CC in the upper segment of the GIT would enhance bioavailability through absorption before reaching the P-gp-rich region in the distal intestine.

## Supporting information

S1 File Detailed procedure for the preparation of CC calibration curve in acetonitrile. (PDF)

S1 Fig CC calibration curve in acetonitrile. (TIF)

S2 Fig **A**. FT-IR spectrum of SE-EE5 after 12 months of storage. **B**. XRD pattern of SE-EE5 after 12 months of storage. **C**. Dissolution profile of SE-EE5 in HCl and phosphate buffer after 12 months of storage.
(TIF)

S1 Graphical abstract (TIF)

## References

[1] Homayun B, Lin X, Choi HJ. Challenges and recent progress in oral drug delivery systems for biopharmaceuticals. Pharmaceutics. 2019 Mar 19;11(3):129. doi: 10.3390/pharmaceutics11030129 30893852 PMC6471246

[2] Baghel S, Cathcart H, O’Reilly NJ. Polymeric amorphous solid dispersions: A review of amorphization, crystallization, stabilization, solid-state characterization, and aqueous solubilization of biopharmaceutical classification system class II drugs. J Pharm Sci. 2016 Sep;105(9):2527–2544. doi: 10.1016/j.xphs.2015.10.008 26886314

[3] Tsume Y, Mudie DM, Langguth P, Amidon GE, Amidon GL. The Biopharmaceutics Classification System: subclasses for in vivo predictive dissolution (IPD) methodology and IVIVC. Eur J Pharm Sci. 2014 Jun 16;57:152–63. doi: 10.1016/j.ejps.2014.01.009 24486482 PMC4112588

[4] Rafiee MH, Abdul Rasool BK. An overview of microparticulate drug delivery system and its extensive therapeutic applications in diabetes. Adv Pharm Bull. 2022 Aug;12(4):730–746. doi: 10.34172/apb.2022.075 36415632 PMC9675914

[5] Abdul Rasool BK, Hussain FN, Bahrainwala IM, Akbar N, Umar S, Kalady SP, et al. Advances in vaccine delivery strategies to promote effective immunization. J Appl Pharm Sci, 2022; 12(05):001–026. doi: 10.7324/JAPS.2022.120501

[6] Kumari L, Choudhari Y, Patel P, Gupta GD, Singh D, Rosenholm JM, et al. Advancement in solubilization approaches: a step towards bioavailability enhancement of poorly soluble drugs. Life. 2023;13(5):1099. doi: 10.3390/life13051099 37240744 PMC10221903

[7] Malkawi R, Malkawi WI, Al-Mahmoud Y, Tawalbeh J. Current trends on solid dispersions: Past, present, and future. Adv Pharmacol Pharm Sci. 2022 Oct 22;2022:5916013. doi: 10.1155/2022/5916013 36317015 PMC9617737

[8] Sharma A, Arora K, Mohapatra H, Sindhu RK, Bulzan M, Cavalu S, et al. Supersaturation-based drug delivery systems: strategy for bioavailability enhancement of poorly water-soluble drugs. Molecules. 2022 May 6;27(9):2969. doi: 10.3390/molecules27092969 35566319 PMC9101434

[9] Zhang X, Xing H, Zhao Y, Ma Z. Pharmaceutical dispersion techniques for dissolution and bioavailability enhancement of poorly water-soluble drugs. Pharmaceutics. 2018;10(3):74. doi: 10.3390/pharmaceutics10030074 29937483 PMC6161168

[10] Rasool BK, Aziz US, Abu-Gharbieh E, Khan SA. Development and evaluation of sustained oral ketorolac tromethamine particulate matrix via bioadhesive chitosan based freeze-dried solid dispersions. Curr Drug Deliv. 2016;13(2):275–86. doi: 10.2174/1567201812666151012114006 26456210

[11] Khan AW, Kotta S, Ansari SH, Sharma RK, Ali J. Enhanced dissolution and bioavailability of grapefruit flavonoid naringenin by solid dispersion utilizing fourth generation carrier. Drug Dev Ind Pharm. 2015 May;41(5):772–9. doi: 10.3109/03639045.2014.902466 24669978

[12] Bhujbal SV, Mitra B, Jain U, Gong Y, Agrawal A, Karki S, et al. Pharmaceutical amorphous solid dispersion: A review of manufacturing strategies. Acta Pharm Sin B. 2021 Aug;11(8):2505–2536. doi: 10.1016/j.apsb.2021.05.014 34522596 PMC8424289

[13] Nikam A, Sahoo PR, Musale S, Pagar RR, Paiva-Santos AC, Giram PS. A Systematic Overview of Eudragit® based copolymer for smart healthcare. Pharmaceutics. 2023 Feb 9;15(2):587. doi: 10.3390/pharmaceutics15020587 36839910 PMC9962897

[14] Candesartan cilexetil. Drug Bank Online. Available at: https://go.drugbank.com/drugs/DB00796 (accessed 2 March 2024).

[15] Hamid S, Beg AE, Muhammad IN, Hassan S, Hassan A, Akram A, et al. Development and validation of HPLC method for the determination of Candesartan in human plasma. Pak J Pharm Sci. 2018 Nov;31(6):2323–2327 30473499

[16] Ghareeb MM, Abdulrasool AA, Hussein AA, Noordin MI. Kneading technique for preparation of binary solid dispersion of meloxicam with poloxamer 188. AAPS PharmSciTech. 2009;10(4):1206–15. doi: 10.1208/s12249-009-9316-0 19862626 PMC2799582

[17] Mohite PB, Khanage SG, Malwade GB. Development and evaluation of cefadroxyl monohydrate solid dispersion via solvent evaporation method: investigating drug-polymer miscibility with advanced characterization. Analytical Chemistry Letters. 2017(7):109–119. doi: 10.1080/22297928.2017.1294991

[18] Chew SL, Modica de Mohac L, Tolulope Raimi-Abraham B. 3D-Printed solid dispersion drug products. Pharmaceutics. 2019 Dec 11;11(12):672. doi: 10.3390/pharmaceutics11120672 31835682 PMC6956082

[19] Abdul Rasool BK, Sammour R. DDSolver software application for quantitative analysis of in vitro drug release behavior of the gastroretentive floating tablets combined with radiological study in rabbits. Curr Drug Deliv. 2022 Aug 6;19(9):949–965. doi: 10.2174/1567201819666220304203014 35249487

[20] Colombo M, Staufenbiel S, Rühl E, Bodmeier R. In situ determination of the saturation solubility of nanocrystals of poorly soluble drugs for dermal application. Int J Pharm. 2017 Apr 15;521(1–2):156–166. doi: 10.1016/j.ijpharm.2017.02.030 28223247

[21] Rafiee MH, Abdul Rasool BK, Haider M, Anbar HS. Oral pioglitazone HCl-loaded solid lipid microparticles: Formulation design and bioactivity studies. J Appl Pharm Sci, 2023;13(02):161–174. doi: 10.7324/JAPS.2023.130218

[22] Sammour RMF, Khan G, Sameer S, Khan S, Zohair T, Saraya S, et al. Development of clindamycin loaded oral microsponges (Clindasponges) for antimicrobial enhancement: In vitro characterization and simulated in vivo studies. Biol Pharm Bull. 2023 Aug 1;46(8):1088–1097. doi: 10.1248/bpb.b23-00099 37245965

[23] Gleiter CH, Mörike KE. Clinical pharmacokinetics of candesartan. Clin Pharmacokinet. 2002;41(1):7–17. doi: 10.2165/00003088-200241010-00002 11825094

[24] Dedroog S, Pas T, Vergauwen B, Huygens C, Van den Mooter G. Solid-state analysis of amorphous solid dispersions: Why DSC and XRPD may not be regarded as stand-alone techniques. J Pharm Biomed Anal. 2020 Jan 30;178:112937. doi: 10.1016/j.jpba.2019.112937 31679845

[25] Abdelwahd A, Abdul Rasool BK. Optimizing and evaluating the transdermal permeation of hydrocortisone transfersomes formulation based on digital analysis of the in vitro drug release and ex vivo studies. Recent Adv Drug Deliv Formul. 2022;16(2):122–144. doi: 10.2174/2667387816666220608115605 35676851 PMC10186384

[26] Sharma N, Singh S. Central composite designed ezetimibe solid dispersion for dissolution enhancement: synthesis and *in vitro* evaluation. Ther Deliv. 2019 Oct;10(10):643–658. doi: 10.4155/tde-2019-0063 31702450

[27] Sester C, Ofridam F, Lebaz N, Gagnière E, Mangin D, Elaissari A. pH-Sensitive methacrylic acid–methyl methacrylate copolymer Eudragit L100 and dimethylaminoethyl methacrylate, butyl methacrylate, and methyl methacrylate tri-copolymer Eudragit E100. Polym Adv Technol. 2020;31:440–450. doi: 10.1002/pat.4780

[28] Wang W, Li M, Yang Q, Liu Q, Ye M, Yang G. The opposed effects of polyvinylpyrrolidone k30 on dissolution and precipitation for indomethacin supersaturating drug delivery systems. AAPS PharmSciTech. 2020 Mar 17;21(3):107. doi: 10.1208/s12249-020-01647-7 32185564

[29] Guzmán ML, Manzo RH, Olivera ME. Eudragit E100 as a drug carrier: the remarkable affinity of phosphate ester for dimethylamine. Mol Pharm. 2012 Sep 4;9(9):2424–33. doi: 10.1021/mp300282f 22808998

[30] Sathigari SK, Radhakrishnan VK, Davis VA, Parsons DL, Babu RJ. Amorphous-state characterization of efavirenz—polymer hot-melt extrusion systems for dissolution enhancement. J Pharm Sci. 2012 Sep;101(9):3456–64. doi: 10.1002/jps.23125 22437488

[31] K S NS, Dengale SJ, Mutalik S, Bhat K. Raloxifene HCl ‐ quercetin co-amorphous system: preparation, characterization, and investigation of its behavior in phosphate buffer. Drug Dev Ind Pharm. 2022 Jun;48(6):227–238. doi: 10.1080/03639045.2022.2104308 35852408

[32] Janssens S. (2009). Review: Physical chemistry of solid dispersions. J Pharm Pharmacol. 2009;61(12):1571–1586. doi: 10.1211/jpp/61.12.0001 19958579

[33] Franco P, De Marco I. The use of poly(n-vinyl pyrrolidone) in the delivery of drugs: A review. Polymers (Basel). 2020 May 13;12(5):1114. doi: 10.3390/polym12051114 32414187 PMC7285361

[34] Yoshida T, Lai TC, Kwon GS, Sako K. pH- and ion-sensitive polymers for drug delivery. Expert Opin Drug Deliv. 2013 Nov;10(11):1497–513. doi: 10.1517/17425247.2013.821978 23930949 PMC3912992

[35] Prasad D, Chauhan H, Atef E. Amorphous stabilization and dissolution enhancement of amorphous ternary solid dispersions: combination of polymers showing drug-polymer interaction for synergistic effects. J Pharm Sci. 2014 Nov;103(11):3511–3523. doi: 10.1002/jps.24137 25196860

[36] Mai Y, Dou L, Yao Z, Madla CM, Gavins FKH, Taherali F, et al. Quantification of p-glycoprotein in the gastrointestinal tract of humans and rodents: methodology, gut region, sex, and species matter. Mol Pharm. 2021 May 3;18(5):1895–1904. doi: 10.1021/acs.molpharmaceut.0c00574 33886332 PMC8289313

[37] Kagan L, Dreifinger T, Mager DE, Hoffman A. Role of p-glycoprotein in region-specific gastrointestinal absorption of talinolol in rats. Drug Metab Dispos. 2010 Sep;38(9):1560–6. doi: 10.1124/dmd.110.033019 20538723

[38] Wang YP, Gan Y, Zhang XX. Novel gastroretentive sustained-release tablet of tacrolimus based on self-microemulsifying mixture: in vitro evaluation and in vivo bioavailability test. Acta Pharmacol Sin. 2011 Oct;32(10):1294–302. doi: 10.1038/aps.2011.90 21927013 PMC4010087

[39] Khalaf MM, Alinejad SS, Sajad O, Abdul Rasool BK. Gastro-retentive drug delivery technologies and their applications with cardiovascular medications. J Popul Ther Clin Pharmacol. 2023;30(5):1–19. doi: 10.47750/jptcp.2023.30.05.001

[40] Rasool BK, Fahmy SA. Development of coated beads for oral controlled delivery of cefaclor: In vitro evaluation. Acta Pharm. 2013 Mar;63(1):31–44. doi: 10.2478/acph-2013-0003 23482311

[41] Anwar W, Dawaba HM, Afouna MI, Samy AM, Rashed MH, Abdelaziz AE. Enhancing the oral bioavailability of candesartan cilexetil loaded nanostructured lipid carriers: In vitro characterization and absorption in rats after oral administration. Pharmaceutics. 2020 Oct 31;12(11):1047. doi: 10.3390/pharmaceutics12111047 33142816 PMC7692391

[42] Cui P, Yin Q, Guo Y, Gong J. Polymorphic crystallization and transformation of candesartan cilexetil. Ind. Eng. Chem. Res. 2012;51(39):12910–12916. doi: 10.1021/ie2024855

[43] Buda V, Baul B, Andor M, Man DE, Ledeţi A, Vlase G, et al. Solid state stability and kinetics of degradation for candesartan-pure compound and pharmaceutical formulation. Pharmaceutics. 2020 Jan 21;12(2):86. doi: 10.3390/pharmaceutics12020086 31972960 PMC7076474

[44] Novakovic D, Isomäki A, Pleunis B, Fraser-Miller SJ, Peltonen L, Laaksonen T, et al. Understanding dissolution and crystallization with imaging: A surface point of view. Mol Pharm. 2018 Nov 5;15(11):5361–5373. doi: 10.1021/acs.molpharmaceut.8b00840 30247922 PMC6221374

[45] Tran TTD, Tran PHL. Molecular interactions in solid dispersions of poorly water-soluble drugs. Pharmaceutics. 2020 Aug 7;12(8):745. doi: 10.3390/pharmaceutics12080745 32784790 PMC7463741

[46] Chabane AF, Bouchal M, Hentabli N, Ayachi HE, Slama, Rezgui F, et al. Investigation of the candesartan cilexetil antihypertensive drug microencapsulation by PLA-PVP K30 biodegradable polymers: Experimental optimization and release kinetics modelling. Can. J. Chem. Eng. 2023, 101(8), 4446. doi: 10.1002/cjce.24811

[47] Paudel A, Ameeduzzafar, Imam SS, Fazil M, Khan S, Hafeez A, et al. Formulation and optimization of candesartan cilexetil nano lipid carrier: In vitro and in vivo evaluation. Curr Drug Deliv. 2017;14(7):1005–1015. doi: 10.2174/1567201813666161230141717 28034361

[48] Lin X, Su L, Li N, Hu Y, Tang G, Liu L, et al. Understanding the mechanism of dissolution enhancement for poorly water-soluble drugs by solid dispersions containing Eudragit® E PO. J Drug Deliv Sci Technol. 2018 Dec;48:328–337. doi: 10.1016/j.jddst.2018.10.008

[49] Bley H, Fussnegger B, Bodmeier R. Characterization and stability of solid dispersions based on PEG/polymer blends. Int J Pharm. 2010 May 10;390(2):165–73. doi: 10.1016/j.ijpharm.2010.01.039 20132875

