# Supplementary material for: Solid dispersion systems for enhanced dissolution of poorly water-soluble candesartan cilexetil: In vitro evaluation and simulated pharmacokinetics studies
Source: PLoS One. 2024 Jun 6;19(6):e0303900. doi: 10.1371/journal.pone.0303900 (PMC11156308; doi:10.1371/journal.pone.0303900)
Supplement: S1 File — (PDF) [file pone.0303900.s001.pdf]

Title: ***Solid dispersion systems for enhanced dissolution of poorly water-soluble candesartan cilexetil: In vitro Evaluation and Simulated Pharmacokinetics Studies***

Supplement 1. Calibration curve of CC

The calibration curve of CC was constructed in acetonitrile (Fig. 1). The stock solution (350 µg/mL) was prepared by dissolving 35 mg of CC powder in acetonitrile in a 100 mL volumetric flask and sonicated for 5 minutes then let to set for another 5 minutes. From this stock solution, a series of dilutions (3.5, 17.5, 35, 70, 105, 140, 175, 210, 245, 280, and 315 µg/mL) of the stock solution were prepared. The elution was accomplished under isocratic pressure, with a mobile phase of acetonitrile and water (55: 45 v/v) with 0.1% TFA. The auto-sampler was set to inject 50 µL from each vial. The process was conducted in triplicate and the readings were calculated as mean ± SD (n=3).

Conc. Mcg/ml	A1	A2	A3	SD
5	0.15	0.16	0.165	0.01
10	0.29	0.298	0.293	0.00
15	0.43	0.427	0.43	0.00
20	0.55	0.591	0.569	0.02
25	0.71	0.707	0.713	0.00
30	0.84	0.849	0.851	0.01
35	0.99	0.985	0.991	0.03
