# Supplementary figures and images for: Solid dispersion systems for enhanced dissolution of poorly water-soluble candesartan cilexetil: In vitro evaluation and simulated pharmacokinetics studies
Source: PLoS One. 2024 Jun 6;19(6):e0303900. doi: 10.1371/journal.pone.0303900 (PMC11156308; doi:10.1371/journal.pone.0303900)

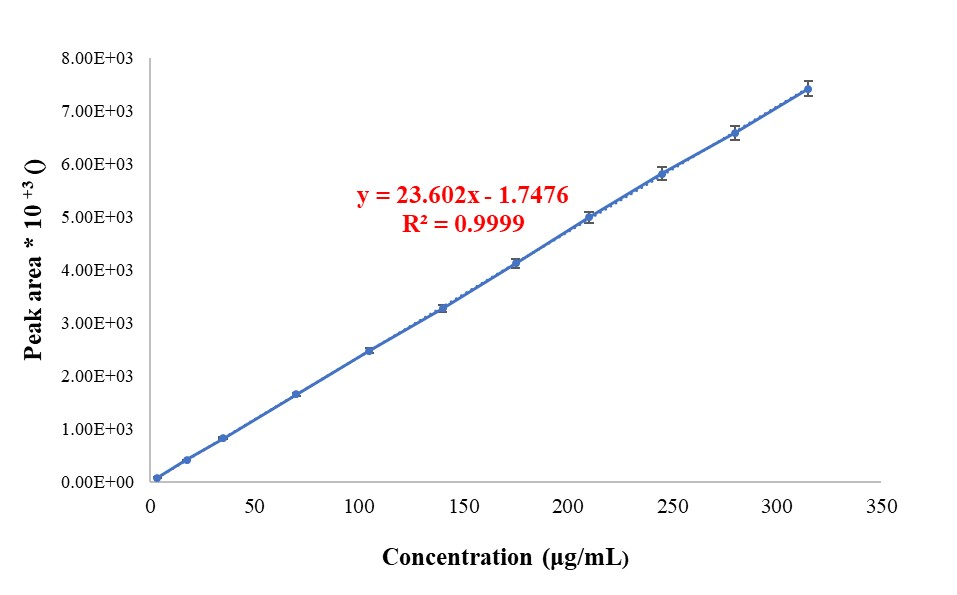

Supplement: S1 Fig — (TIF) [file pone.0303900.s002.tif]

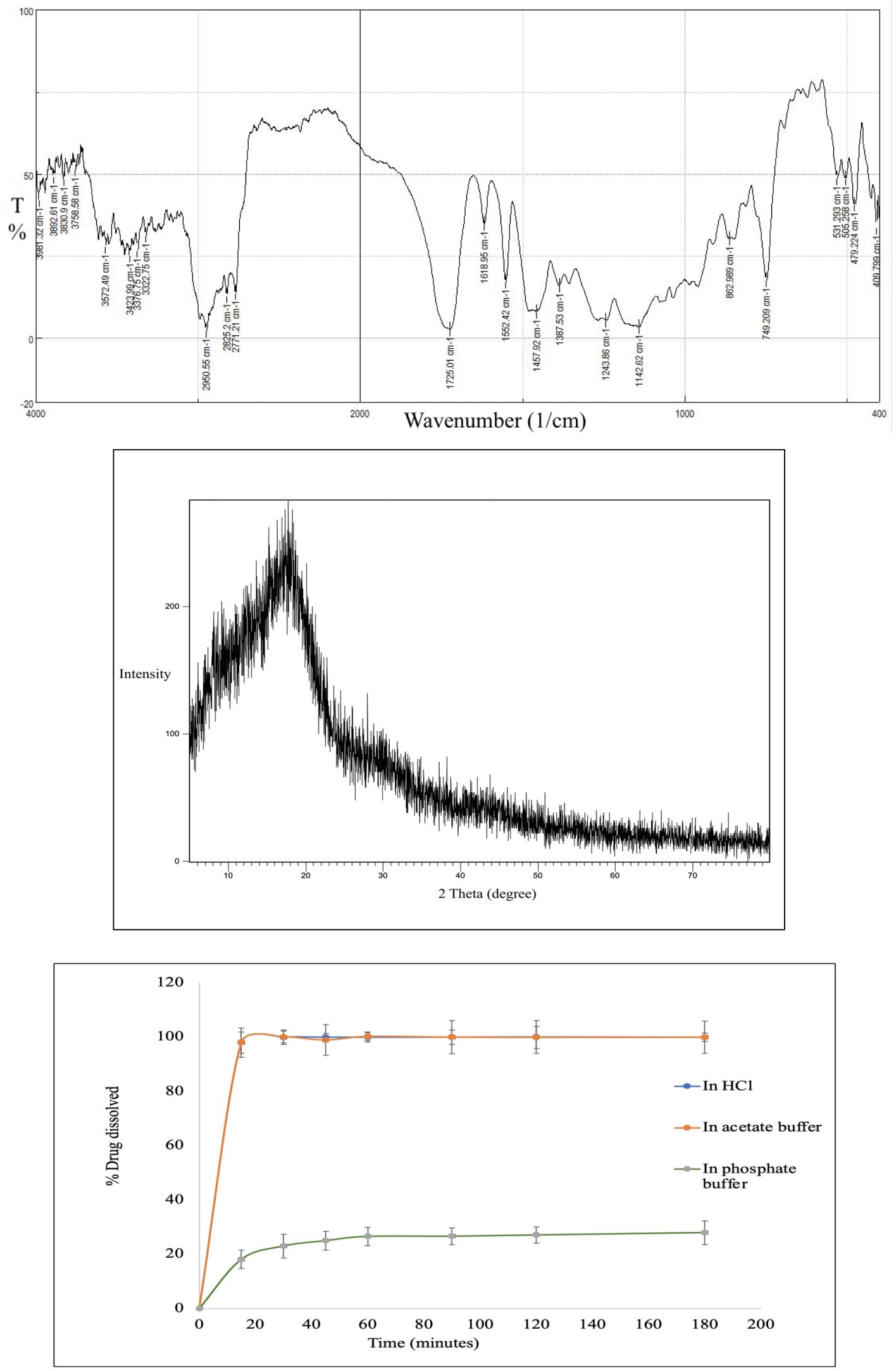

Supplement: S2 Fig — A. FT-IR spectrum of SE-EE5 after 12 months of storage. B. XRD pattern of SE-EE5 after 12 months of storage. C. Dissolution profile of SE-EE5 in HCl and phosphate buffer after 12 months of storage. (TIF) [file pone.0303900.s003.tif]

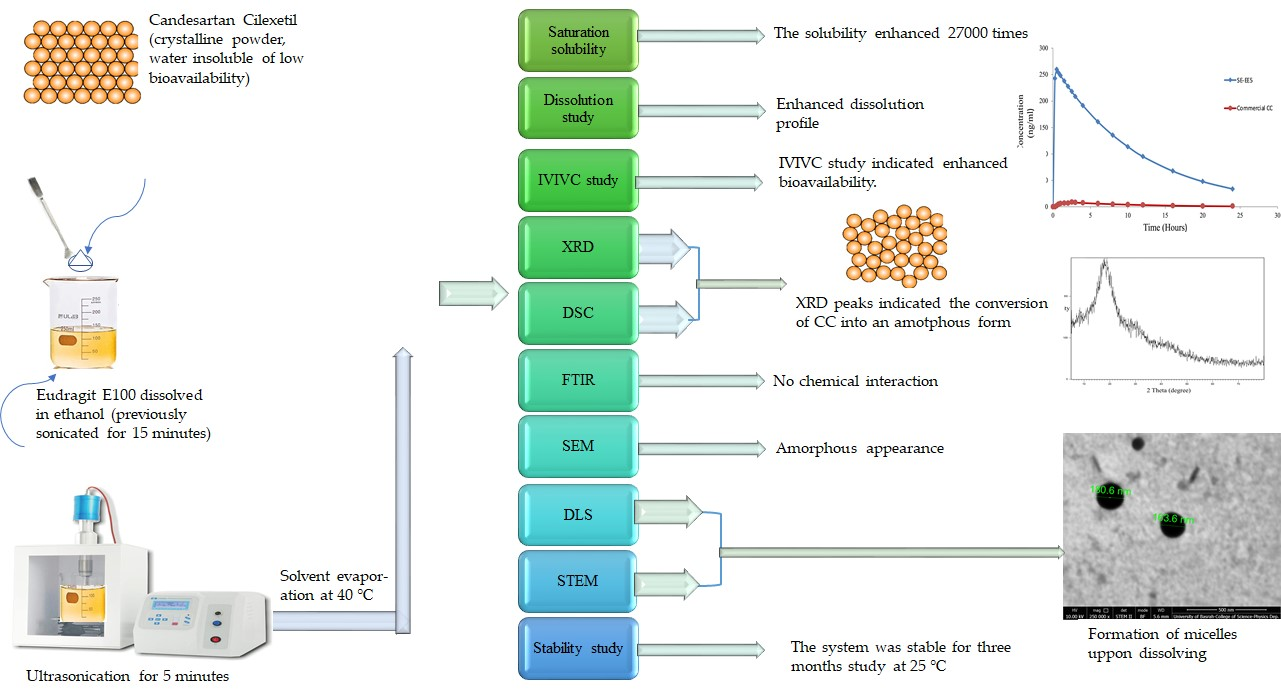

Supplement: S1 Graphical abstract — (TIF) [file pone.0303900.s004.tif]
